# Identification and expression analysis of calcium-dependent protein kinase family in oat (*Avena sativa* L.) and their functions in response to saline-alkali stresses

**DOI:** 10.3389/fpls.2024.1395696

**Published:** 2024-10-10

**Authors:** Ya-nan Li, Chunyan Lei, Qian Yang, Xiao Yu, Siming Li, Yan Sun, Chunli Ji, Chunhui Zhang, Jin-ai Xue, Hongli Cui, Runzhi Li

**Affiliations:** ^1^ College of Agriculture, Institute of Molecular Agriculture and Bioenergy, Shanxi Agricultural University, Jinzhong, Shanxi, China; ^2^ Key Laboratory of Coastal Biology and Bio-Resource Utilization, Yantai Institute of Coastal Zone Research, Chinese Academy of Sciences, Yantai, Shandon, China

**Keywords:** oat (*Avena sativa* L.), saline-alkali stresses, calcium-dependent protein kinase (CDPK*)*, *Chlamydomonas reinhardtii*, genetic transformation, Na+/ H+ antiporter 1 (NHX1)

## Abstract

Calcium-dependent protein kinases (CDPKs) serve as calcium ion sensors and play crucial roles in all aspects of plant life cycle. While *CDPK* gene family has been extensively studied in various plants, there is limited information available for *CDPK* members in oat, an important cereal crop worldwide. Totally, 60 *AsCDPK* genes were identified in oat genome and were classified into four subfamilies based on their phylogenetic relationship. The members within each subfamily shared similar gene structure and conserved motifs. Collinearity analysis revealed that *AsCDPK* gene amplification was attributed to segmental duplication events and underwent strong purifying selection. *AsCDPK* promoters were predicted to contain *cis*-acting elements associated with hormones, biotic and abiotic stresses. *AsCDPK* gene expressions were induced by different salt stresses, exhibiting stress-specific under different salt treatments. Moreover, overexpression of *AsCDPK26* gene enhanced salt resistance in *C. reinhardtii*, a single-cell photoautotrophic model plants. Further analysis revealed a significant correlation between *AsCDPK26* and Na^+^/H^+^ antiporter 1 (*p*<0.05), suggesting that AsCDPK26 may interact with ion transporter to modulate salt resistance. These results not only provide valuable insights into *AsCDPK* genes in response to different salt stresses, but also lay the foundation to mine novel candidates for improving salt tolerance in oat and other crops.

## 1 Introduction

Plants have developed a series of effective defense mechanisms to cope with complex and variable environmental conditions. Signal transduction pathways are crucial in these processes (Trewavas and Malhó, 1998). Calcium ion (Ca^2+^), as ubiquitous secondary messengers, play a vital role in signal transduction pathways. Moreover, other molecules also play significant roles in plant stress responses, including small lipid molecules, cAMP (cyclic adenosine monophosphate), and cGMP (cyclic guanosine monophosphate) (Trewavas and Gilroy, 1991; Zhao et al., 2021a). However, free Ca^2+^ has the widest range of action. When plants encounter abiotic stresses such as temperature, light, salt, and osmotic stress, certain calcium receptors or calcium binding proteins can detect the changes in Ca^2+^ concentration and recognize the calcium signals. These proteins then transmit signals to downstream components through phosphorylation, leading to the expression of the related genes (Harmon et al., 2001). This mechanism helps plants to cope with environmental stress and enhance their resistance. Plant cells contain a variety of calcium-binding proteins, such as calmodulin (CaM), calcineurin B-like proteins (CBLs), and calcium-dependent protein kinase (CDPKs or CPKs) (Cheng et al., 2002).

CDPK can sense and respond to changes in Ca^2+^ concentration, thereby regulating various crucial cell signaling processes. Unlike other Ca^2+^ sensors, CDPK has both Ca^2+^ binding and kinase activities, allowing it to bind Ca^2+^ directly and convert the signals into phosphorylation events downstream (Boudsocq and Sheen, 2013). The typical CDPK protein structure consists of four main parts: the N-terminal variable region, the serine/threonine (Ser/Thr) kinase domain, the auto-inhibitory domain (also known as the connection domain), and the calmodulin-like binding domain (Cheng et al., 2002). *CDPKs* have been identified in several plant species, including *Arabidopsis thaliana* (Cheng et al., 2002), *Triticum aestivum* (Liu et al., 2023b), *Oryza sativa* (Asano et al., 2005), and *Zea mays* (Kong et al., 2013).

*CDPK* plays a crucial role in regulating plant growth and responses to various stresses (Kong et al., 2013). Studies have shown that *CDPK* members within the same plant species exhibit functional diversity, and there are quantitative and functional differences among *CDPKs* from different plants. For example, in *Arabidopsis*, the *AtCPK1* mutants display sensitivity to salt and drought stress, whereas overexpression of *AtCPK1* significantly enhances resistance to these stresses (Huang et al., 2018). Similarly, other *CDPK* genes in *Arabidopsi*s, such as *AtCPK3/4/11*, also function as positive regulators in response to salt and drought stress (Mehlmer et al., 2010; Zhu et al., 2007). Conversely, *AtCPK23* acts as a negative regulator in plant responses to drought and salt stress. The T-DNA insertion mutant *cpk23* exhibits significantly enhanced tolerance to drought and salt stress, whereas the overexpression line is more sensitive to these stresses (Ma and Wu, 2007). In rice, over-expression of *OsCDPK7* enhanced induction of some stress-responsive genes in response to salinity/drought, but not to cold stress (Saijo et al., 2000). *OsCDPK13* and *OsCPK17* are considered as important signaling components in rice response to cold stress (Almadanim et al., 2017; Komatsu et al., 2007). As a direct ‘sensor’ of Ca^2+^, CDPK can directly or indirectly regulate crucial downstream target proteins, including transcription factors (Yang et al., 2021), ion channels (Latz et al., 2013), or other proteins related to signaling pathways (Zou et al., 2015; Atif et al., 2019). For example, *AtCPK8* plays an important role in regulating catalase CAT3 activity via phosphorylation, which is part of ABA-mediated stomatal regulation under drought stress (Zou et al., 2015). In peppers, *CaWRKY27b* is phosphorylated at Ser137 by *CaCDPK29*, leading to its translocation from the cytoplasm to the nucleus. This physical interaction enhances the function of *CaWRKY40* as a positive regulator of anti-RSI immunity and heat tolerance in peppers (Yang et al., 2021).

Soil salinization is a critical environmental stressor that affects seed germination, crop growth, and productivity, posing a threat to agricultural yields and ecological security globally (Zhou et al., 2024). High sodium concentrations in saline soils limit plant water uptake and nutrient absorption (Zhao et al., 2021b). Water deficiency and nutritional imbalances induce both osmotic stress and ionic stress. These primary stresses can result in oxidative stress, which in turn triggers a cascade of secondary stresses (Zhu, 2002). More than 900 million hectares of the world’s land are affected by soil salinization, and there are no effective practices to control its spread (Fang et al., 2021). It is projected that the global population will reach 9.7 billion by 2050, and the surge in food demand due to this population growth compels us to develop saline areas for cultivation and to breed salt-tolerant crops (Gu et al., 2021). Oat (*Avena sativa* L.) cultivated extensively in the word is one of the most widely consumed cereal grains, with high nutritional value and potential health benefits for human and animals (Sánchez-Martín et al., 2014). As a traditional grain crop in saline areas, oat has less stringent cultivation soil requirements, exhibiting higher salt tolerance than wheat, rice, and other staple food crops (Han et al., 2014). These attributes render oats a promising crop for the improvement of saline-alkali lands. Therefore, identification and characterization of the salt tolerant genes from oat are urgently needed for breeding of elite oat varieties. As direct sensors of Ca^2+^, CDPKs play an important role in regulating plant growth and various stress responses. However, the *CDPK* gene family, especially those associating with salt tolerance, has not been well studied in oat. Understanding the molecular mechanisms of *CDPK* involvement in salt resistance or tolerance in oat will provide insight on how to improve plant salt stress resistance and is a critical step in improving agricultural productivity and food security. In this study, *AsCDPK* members in oat were systematically characterized, including their physicochemical properties, chromosome distribution, gene structure and duplication, synteny, and phylogenetic relationship. The expression patterns of *AsCDPK* genes under different salt stresses were examined using RNA-seq data and further verified by RT-qPCR analysis. Moreover, possible interacting ion transporters were predicted through correlation analysis. Particularly, *AsCDPK26* gene was cloned and functionally characterized by heterogenous overexpression in *Chlamydomonas reinhardtii* (CC849 strain), a single-cell photoautotrophic model plant, followed by phenotypic analysis of the transgenic lines under different salt stresses. And the correlation between AsCDPK26 and ion transporters in *Chlamydomonas* was also further verified by RT-qPCR analysis. This study provided valuable information for further exploration of the functions of oat *AsCDPK* genes in responses to salinity and other abiotic stresses, highlighting *AsCDPK26* as a suitable target gene in plant biotechnology for improvement of stress resistance in crops.

## 2 Materials and methods

### 2.1 Experimental materials and treatments

The oat variety used in this study is CEav5651. This experiment was performed in the greenhouse, and the oat plants underwent hydroponic trials. Equal sized and healthy oat seeds were selected and sterilized using 3% NaClO for 10 min, followed by three rinses with distilled water. The seeds were kept on moist filter paper for germination. Incubation conditions were 16 h of light at 25°C, 8 h of darkness at 20°C, and humidity of 80%. After the seeds germinate fully, the seedlings were transplanted in a 96-hole water boxes. These boxes are filled with 1/2 Hoagland nutrient solution, with 48 seeds per box, cultivated in the greenhouse. The nutrient solution was replaced every 2 days. The greenhouse cultivation conditions consisted of 16 h of light at 25-30°C and 8 h of darkness at 15-18°C. When seedlings grew to two-leaf stage, the plants were treated with salt for 48 h. Saline and/or alkali treatments included three salt conditions (neutral salt stress (NaCl), alkaline salt stress (NaHCO_3_), and mixed salt-alkali stress (1:1 ratio of NaCl and NaHCO_3_)), two levels (100 and 200 mmol·L^-1^), and three replicates. The medium without saline and/or alkali treatment was the control group. Fresh aboveground tissues were immediately frozen in liquid nitrogen and stored at –80°C for transcriptome analysis.

### 2.2 Identification of *AsCDPK* gene family and analysis of basic parameters

The amino acid sequences of *Arabidopsis* AtCDPKs were downloaded from Phytozomev13 (https://phytozome-next.jgi.doe.gov/). The coding sequences (CDS) and the corresponding amino acid sequences were obtained from the oat genome database (https://wheat.pw.usda.gov/GG3/graingenes-downloads/pepsico-oat-oc3098-v2-files-2021). The *AtCDPK* sequences were used as queries to identify candidate *AsCDPK* genes by using BLASTP with an E-value less than e^-10^. Sequences containing both the Pkinase domain and EF-hand motif were filtered using the Interpro tool (http://www.ebi.ac.uk/interpro/). The composition of the identified candidate proteins was further verified in SMART databases (http://smart.embl-heidelberg.de/), while the sequences with errors, shorter length (<100 aa), and containing incomplete Ser/Thr kinase domain were eliminated. Amino acid number (aa), isoelectric point (pI), molecular weight (MW), and other parameters of AsCDPK proteins were predicted using the ExPASy Proteomics Server (https://web.expasy.org/protparam/) (Linghu et al., 2023). WoLF PSORT (https://wolfpsort.hgc.jp) (Yu et al., 2024) predictor was used to predict the subcellular localization of AsCDPK proteins, and the N-terminal myristoylation site was predicted using the GPS-Lipid 1.0 program with default settings and high threshold (Zhao et al., 2021a).

### 2.3 Multiple sequence alignments and phylogenetic analysis

Multiple alignments of CDPK protein sequences from oat, *Arabidopsis*, and rice were performed using the Clustal W program, with default parameters implemented in MEGA7.0 software (Chen et al., 2023). The phylogenetic tree was constructed by MEGA7.0 with the neighbor-joining method based on the sequence alignment results. The bootstrap value was set to 1000.

### 2.4 Chromosome mapping, gene structure and selection pressure analysis

The chromosomal distribution of the *AsCDPK* genes was visualized using TBtools software (Chen et al., 2020) based on the list of *AsCDPK* members and the oat genome annotation file. Synteny analysis was performed using the multicollinear scanning tool package (MCScanX) to identify the collinearity pattern of *CDPKs* among oat (Wang et al., 2023). Additionally, the values of nonsynonymous (*Ka*) and synonymous (*Ks*) substitution were calculated for fragment duplication gene pairs. The gene structure was displayed by comparing the coding sequence and the corresponding genomic DNA sequence using the gene structure display server tool (http://gsds.cbi.pku.edu.cn/).

### 2.5 Conserved motif and functional domain analysis

The conserved motifs were visualized using TBtools software, and each protein’s conserved motifs were analyzed using the MEME program (https://meme-suite.org) (Liu et al., 2023a). The maximum number of motifs was set to 20, and the optimal motif width was set to ranged from 10 to 100 amino acids. The remaining parameters were set to their default values. The Interpro (www.ebi.ac.uk/interpro) and SMART (smart.embl-heidelberg.de) databases were used to identify the functional domains and key sites in AsCDPKs. Subsequently, the TBtools toolkit (https://github.com/CJ-Chen/TBtools) was used to draw the diagram.

### 2.6 Analysis of *cis*-acting elements in the promoters of *AsCDPK* genes

The 2,000 bp promoter sequence upstream of each *AsCDPK* transcription start point was extracted from the oat genome database. The sequence was analyzed using the PlantCare online software (http://bioinformatics.psb.ugent.be/webtools/PlantCare/html/) to predict potential *cis*-acting regulatory elements (Cui et al., 2024).

### 2.7 *AsCDPK* gene expression profiles and RT- qPCR analysis

The expression patterns of *AsCDPK* genes were analyzed using a transcriptome database derived from oat seedlings subjected to various salt treatments. The expression levels were measured using the fragments per kilobase of exon model per million mapped fragments (FPKM) method. Total RNA was extracted from oat aboveground parts using Trizol (Simgen, Hangzhou, China). The cDNA was synthesized by using the All-in-One First-Strand cDNA Synthesis Super Mix for qPCR Kit (One-Step gDNA Removal; TransGen, Beijing, China) according to specifications. PCR identification was performed using the gene-specific primers listed in **Supplementary Table S1** and the reaction system described in **Supplementary Table S2**. RT-qPCR detection was conducted using a Takara kit, with each sample repeated three times. The relative expression of genes was calculated using the 2^-ΔΔCt^ method. The PCR amplification conditions were set as follows: (1) 95°C for 10 min; (2) 95°C for 15 s, 60°C for 1 min for 39 cycles.

### 2.8 Genetic transformation and identification

*C. reinhardtii* CC849 used as the host in this study was obtained from Hu Zhangli’s research group at Shenzhen University, China. The constitutive expression vector pHR13-*AsCDPK26* contained the ORF of *AsCDPK26* gene and the hygromycin resistance gene (*Hyg*). The plasmid pHR13-*AsCDPK26* was extracted from the puncture bacteria using the SIMGEN plasmid DNA mini kit (Simgen, Hangzhou, China), and then used for genetic transformation of *C. reinhardtii* by the glass strain transformation method (Wang et al., 2017a). The algal cells were cultured in 50 mL fresh Tris-Acetate-Phosphate (TAP) medium under white light and selected by 10 mg L^−1^ hygromycin in TAP agar medium plates. Genomic DNA was extracted from CC849, empty vector (EV) and transgenic strains using the cetyltrimethylammonium bromide (CTAB) method. The positive transgenic algal strains screened by hygromycin were verified using genomic DNA PCR analysis. The primers used were *AsCDPK26-*F1 and *AsCDPK26-*R1 (**Supplementary Table S1**). Total RNA was extracted using Trizol kit (Simgen, Hangzhou, China) and cDNA synthesis performed using the All-in-One First-Strand cDNA Synthesis Super Mix for qPCR Kit (One-Step gDNA Removal; TransGen, Beijing, China). Transgenic algal strains were verified using cDNA PCR analysis with the primers *AsCDPK26*-F2 and *AsCDPK26*-R2 (**Supplementary Table S1**). To quantitatively detect *AsCDPK26* gene expression in both wild type and transgenic strains, RT-qPCR was performed with Bio-Rad CFX Connect Optics Module (the primers was listed in **Supplementary Table S1**). The PCR amplification procedure were as follows: (1) 95°C for 10 min; (2) 95°C for 15 s, 58°C for 1 min for 39 cycles. The relative expression of genes was calculated using the 2^-ΔΔCt^ program. The reaction were conducted with three biological replicates and three technical replicates.

### 2.9 Salt treatment, cell viability determination and pigment measurement

Algal growth study was performed for 96 h in sterile TAP medium after saline and/or alkali treatments. The initial optical density (OD_680_) was adjusted to approximately 0.3, and the samples were placed in a continuous light incubator at 25°C and 95 μmol·m^-2^·s^-1^. The growth characteristics of the algal cells were measured using the 680 nm optical density method. Saline and/or alkali treatments included three salt conditions (neutral salt stress (NaCl), alkaline salt stress (NaHCO_3_), and mixed salt-alkali stress (1:1 ratio of NaCl and NaHCO_3_)), three levels (100, 200, and 300 mmol·L^-1^), and three replicates. The medium without saline and/or alkali treatment was the control group, and culture condition was the same with single strain culture. Manual shaking was performed five times daily to maintain the uniform distribution of culture and medium components. On the final day, a sample of the microalgae was taken and filtered using a 0.45 μm microporous membrane to remove any remaining inorganic salts. The membrane was then dried at 60°C until the weight remained constant to calculate cell mass. All reported data represent the averages of three biological replicates. Total chlorophyll contents in algal cells were measured using spectrophotometry (Hamed et al., 2017). The chlorophyll fluorescence intensity was measured using a Handy-PEA chlorophyll fluorometer (Hansatech Instruments Ltd, UK).

### 2.10 Statistical analysis

All data were analyzed by Microsoft Excel 2010 and GraphPad Prism 8.0.2 (GraphPad Software, San Diego, USA). Experiments were carried out with biological replicates, and data were presented as the mean with standard deviation (mean ± SD). SPSS26.0 (SPSS, USA) was used for statistical analysis by one-way analysis of variance (ANOVA). For all of data analysis, a *p*-value<0.01 was considered as highly significant difference while a *p*-value<0.05 represents statistically significant.

## 3 Results

### 3.1 Identification of *AsCDPK* gene family members and their protein physicochemical properties

A total of 60 *AsCDPK* genes were identified in oat genome, using the known *Arabidopsis* AtCDPK proteins as query sequences. These *AsCDPK* genes were named *AsCDPK1*~*AsCDPK60* (**Table 1**), respectively. The encoded amino acid (aa) lengths of these *AsCDPK* genes ranged from 511 to 1,198 aa, with corresponding molecular weights (MW) varied from 56,453.08 to 135,417.29 bp. The majority of AsCDPK members had isoelectric points (pI) values below 7.0, indicating their acidic nature, except for AsCDPK22/41/46/47/49/51/55/56. Of the 55 AsCDPKs, most contained four EF-hands. However, AsCDPK24/30/58/59/60 had three EF-hands. All AsCDPKs contained the predicted N-terminal myristoylation sites, except for AsCDPK2/14/22/24/58/59/60. In addition, subcellular localization prediction indicated that AsCDPKs were primarily located in the cytoplasm, chloroplasts, and mitochondria, with a smaller numbers found in the nucleus and endoplasmic reticulum.

**Table 1:** Information of *AsCDPK* genes in oat.

Gene name	Gene ID	No. of aa	MW (kD)	PI	No. of EF Hands	N-myrist^a^	Subcellular localization^b^
*AsCDPK1*	AVESA.00001b.r1.2Ag0000636.1	572	62 666.47	5.13	4	Y	chlo: 5, vacu: 4, E.R.: 2,
*AsCDPK2*	AVESA.00001b.r1.4Ag0001693.1	624	69 004.57	6.06	4	N	chlo: 5, vacu: 5, nucl: 1
*AsCDPK3*	AVESA.00001b.r1.2Cg0002505.1	554	60 899.19	5.50	4	Y	mito: 8.5, chlo_mito: 6, chlo: 2.5
*AsCDPK4*	AVESA.00001b.r1.6Cg0003063.1	546	60 434.66	5.29	4	Y	mito: 9.5, chlo_mito: 7, chlo: 3.5
*AsCDPK5*	AVESA.00001b.r1.7Cg0000523.1	636	69 801.43	5.82	4	Y	vacu: 6, chlo: 4, golg: 3
*AsCDPK6*	AVESA.00001b.r1.7Cg0000520.1	589	64 638.63	5.36	4	Y	chlo: 5, vacu: 5, golg: 2
*AsCDPK7*	AVESA.00001b.r1.2Dg0001748.1	555	61 108.48	5.56	4	Y	mito: 9.5, chlo_mito: 6, cyto: 2
*AsCDPK8*	AVESA.00001b.r1.2Dg0003120.1	568	62 426.2	5.09	4	Y	chlo: 4, vacu: 4, E.R.: 2
*AsCDPK9*	AVESA.00001b.r1.4Dg0002013.1	638	70 272.01	6.09	4	Y	vacu: 6, chlo: 4, golg: 2
*AsCDPK10*	AVESA.00001b.r1.6Dg0000850.1	549	60 588.88	5.48	4	Y	mito: 8.5, chlo_mito: 7, chlo: 4.5
*AsCDPK11*	AVESA.00001b.r1.2Ag0001803.1	555	61 106.5	5.57	4	Y	mito: 8.5, chlo_mito: 6, chlo: 2.5
*AsCDPK12*	AVESA.00001b.r1.4Ag0000684.1	511	56 469.08	5.20	4	Y	chlo: 11, nucl: 1, mito: 1
*AsCDPK13*	AVESA.00001b.r1.4Ag0002412.1	543	61 167.48	5.16	4	Y	cyto: 7, E.R.: 5, nucl: 1
*AsCDPK14*	AVESA.00001b.r1.5Ag0001479.1	528	57 850.85	5.59	4	N	chlo: 4, cyto: 3, plas: 3
*AsCDPK15*	AVESA.00001b.r1.6Ag0001120.1	552	60 818.07	5.43	4	Y	mito: 8.5, chlo_mito: 7, chlo: 4.5
*AsCDPK16*	AVESA.00001b.r1.4Cg0000976.1	543	61 083.51	5.24	4	Y	cyto: 6, E.R.: 5, nucl: 1
*AsCDPK17*	AVESA.00001b.r1.7Cg0001593.1	511	56 481.13	5.20	4	Y	chlo: 11, mito: 1, plas: 1
*AsCDPK18*	AVESA.00001b.r1.1Dg0003388.1	543	61 156.45	5.12	4	Y	cyto: 6, E.R.: 5, nucl: 1
*AsCDPK19*	AVESA.00001b.r1.3Dg0001914.1	516	57 400.31	5.34	4	Y	nucl: 3.5, chlo: 3, plas: 3
*AsCDPK20*	AVESA.00001b.r1.4Dg0001000.1	511	56 453.08	5.20	4	Y	chlo: 12, mito: 1, plas: 1
*AsCDPK21*	AVESA.00001b.r1.1Ag0003574.1	526	59 483.71	6.24	4	Y	cyto: 9, nucl: 2, chlo: 1
*AsCDPK22*	AVESA.00001b.r1.1Ag0002101.1	663	74 437.07	9.02	4	N	chlo: 13, mito: 1
*AsCDPK23*	AVESA.00001b.r1.2Ag0000104.1	537	59 886.15	5.68	4	Y	chlo: 8, nucl: 2, cyto: 1
*AsCDPK24*	AVESA.00001b.r1.3Ag0001628.1	597	66 796.8	5.85	3	N	cyto: 7, nucl: 4, chlo: 1
*AsCDPK25*	AVESA.00001b.r1.4Ag0002698.1	516	57 416.31	5.34	4	Y	chlo: 3, plas: 3, nucl: 2.5
*AsCDPK26*	AVESA.00001b.r1.2Cg0000672.1	536	59 760.99	5.63	4	Y	chlo: 5, nucl: 3, cyto: 2
*AsCDPK27*	AVESA.00001b.r1.7Cg0001726.1	532	58 683.7	5.55	4	Y	cyto: 10, nucl: 3, cysk: 1
*AsCDPK28*	AVESA.00001b.r1.1Dg0002135.1	530	59 901.18	6.26	4	Y	cyto: 10, chlo: 1, nucl: 1
*AsCDPK29*	AVESA.00001b.r1.2Dg0002567.1	537	59 870.11	5.68	4	Y	chlo: 8, nucl: 2, cyto: 1
*AsCDPK30*	AVESA.00001b.r1.3Dg0001187.1	509	57 234.14	5.74	3	Y	mito: 10, chlo: 3, nucl: 1
*AsCDPK31*	AVESA.00001b.r1.1Ag0002914.1	551	60 895.32	6.25	4	Y	cyto: 6.5, cyto_nucl: 4, E.R.: 2
*AsCDPK32*	AVESA.00001b.r1.1Ag0001427.1	548	60 689.12	6.29	4	Y	cyto: 6.5, cyto_nucl: 4, E.R.: 2
*AsCDPK33*	AVESA.00001b.r1.2Ag0001721.1	537	59 746.38	5.92	4	Y	cyto: 7, nucl: 4, chlo: 1
*AsCDPK34*	AVESA.00001b.r1.3Ag0002268.1	542	60 666.33	6.38	4	Y	cyto: 6.5, cyto_nucl: 4, E.R.: 2
*AsCDPK35*	AVESA.00001b.r1.4Ag0001825.1	533	60 207.64	6.19	4	Y	chlo: 4, plas: 3, E.R.: 2
*AsCDPK36*	AVESA.00001b.r1.2Cg0002423.1	534	59451.08	5.87	4	Y	nucl: 6, cyto: 5, chlo: 1
*AsCDPK37*	AVESA.00001b.r1.3Cg0002463.1	542	60 649.39	6.50	4	Y	cyto: 6.5, cyto_nucl: 4, E.R.: 2
*AsCDPK38*	AVESA.00001b.r1.1Dg0001468.1	547	60 648.07	6.29	4	Y	cyto: 6.5, cyto_nucl: 4, E.R.: 2
*AsCDPK39*	AVESA.00001b.r1.2Dg0001661.1	537	59 684.27	5.92	4	Y	cyto: 7, nucl: 5, chlo: 1
*AsCDPK40*	AVESA.00001b.r1.4Dg0002141.1	533	60 191.64	6.19	4	Y	chlo: 4, plas: 3, E.R.: 2
*AsCDPK41*	AVESA.00001b.r1.4Ag0001166.1	587	65 159.39	9.07	4	Y	chlo: 14
*AsCDPK42*	AVESA.00001b.r1.4Ag0001829.1	537	60 919.32	6.22	4	Y	cyto: 8, chlo: 2, mito: 2
*AsCDPK43*	AVESA.00001b.r1.6Ag0002165.1	537	60 666.47	6.80	4	Y	vacu: 5, cyto: 2.5, chlo: 2
*AsCDPK44*	AVESA.00001b.r1.2Cg0000500.1	536	60 498.27	7.00	4	Y	vacu: 6, chlo: 5, nucl: 1
*AsCDPK45*	AVESA.00001b.r1.7Cg0000381.1	533	60 165.6	6.19	4	Y	E.R.: 4, chlo: 3, plas: 3
*AsCDPK46*	AVESA.00001b.r1.7Cg0001047.1	578	64 268.35	8.63	4	Y	chlo: 6, vacu: 3, plas: 2
*AsCDPK47*	AVESA.00001b.r1.2Dg0002401.1	794	88 946.92	7.85	4	Y	vacu: 7, golg: 3, chlo: 1
*AsCDPK48*	AVESA.00001b.r1.3Dg0001812.1	517	57 775.93	6.19	4	Y	cyto: 6.5, cyto_nucl: 4, E.R.: 2
*AsCDPK49*	AVESA.00001b.r1.4Dg0001473.1	587	65 187.44	9.07	4	Y	chlo: 14
*AsCDPK50*	AVESA.00001b.r1.5Dg0001148.1	559	62 636.59	5.75	4	Y	mito: 8, chlo: 5, golg: 1
*AsCDPK51*	AVESA.00001b.r1.1Ag0002412.1	517	58 172.59	8.96	4	Y	mito: 5.5, chlo: 4, nucl: 4
*AsCDPK52*	AVESA.00001b.r1.5Ag0001519.1	559	62 635.61	5.75	4	Y	mito: 8, chlo: 5, golg: 1
*AsCDPK53*	AVESA.00001b.r1.5Ag0002848.1	551	61 890.56	6.01	4	Y	mito: 4, chlo: 3, nucl: 2
*AsCDPK54*	AVESA.00001b.r1.5Cg0002481.1	549	61 788.42	5.82	4	Y	chlo: 3, mito: 3, vacu: 3
*AsCDPK55*	AVESA.00001b.r1.6Cg0001428.1	517	58 082.45	8.96	4	Y	chlo: 6, mito: 4.5, nucl: 3
*AsCDPK56*	AVESA.00001b.r1.2Dg0000664.1	518	58 259.67	8.96	4	Y	chlo: 7, mito: 3.5, nucl: 3
*AsCDPK57*	AVESA.00001b.r1.5Dg0002397.1	551	61 862.55	6.12	4	Y	mito: 4, chlo: 3, nucl: 2
*AsCDPK58*	AVESA.00001b.r1.3Ag0001659.1	1 198	135 417.29	5.09	3	N	nucl: 8, cyto: 5, cysk: 1
*AsCDPK59*	AVESA.00001b.r1.3Cg0000800.1	1 154	130 325.79	4.97	3	N	cyto: 9, nucl: 4, cysk: 1
*AsCDPK60*	AVESA.00001b.r1.3Dg0001210.1	519	57 670.97	5.50	3	N	chlo: 7, cyto: 4, nucl: 2

MW, Molecular weight; PI, Isoelectric point. ^a^The myristoylation site was predicted by Myristoylator program in ExPASy (http://web.expasy.org/myristoylator/). ^b^chlo, Chloroplast; cysk, Cytoskeleton; cyto ,Cytoplasmic; E.R., Endoplasmic reticulum; mito, Mitochondrial; nucl, Nuclear; pero, Peroxysome; plas, Plasma membrane; vacu, Vacuole.

### 3.2 Phylogenetic analysis and chromosomal distribution of *AsCDPK* genes

**Figure 1:**
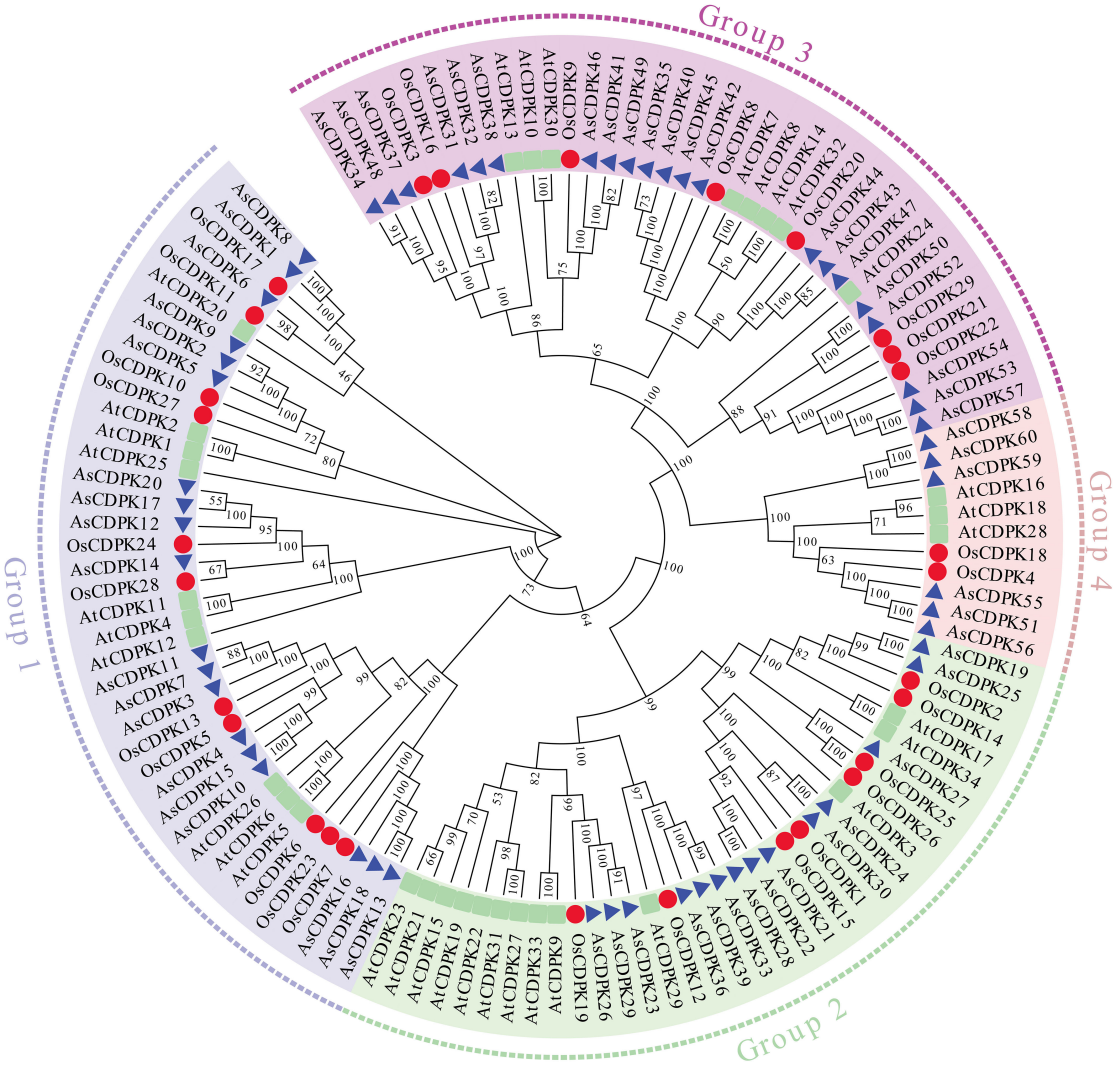
Phylogenic tree of CDPK proteins from oat, rice and *Arabidopsis*.

**Figure 2:**
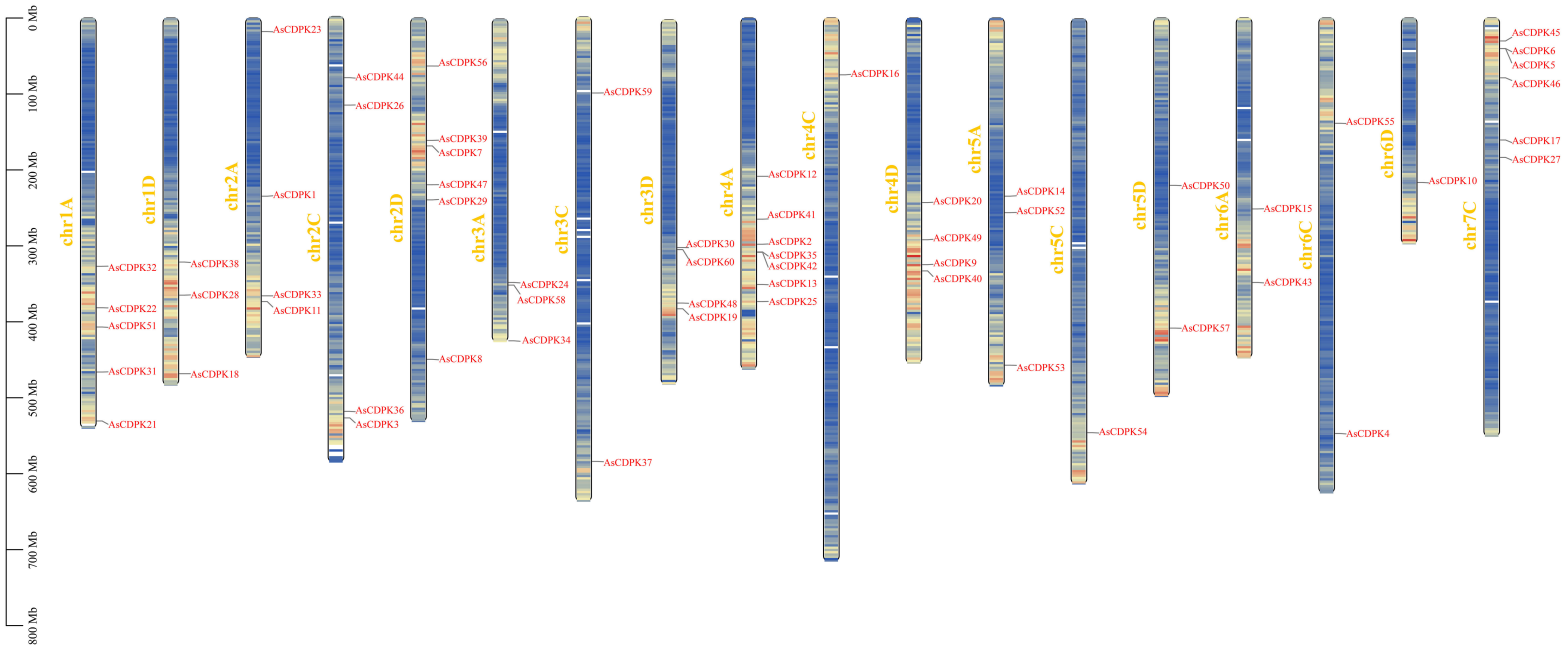
Chromosomal locations of *AsCDPK* genes in oat.

**Figure 3:**
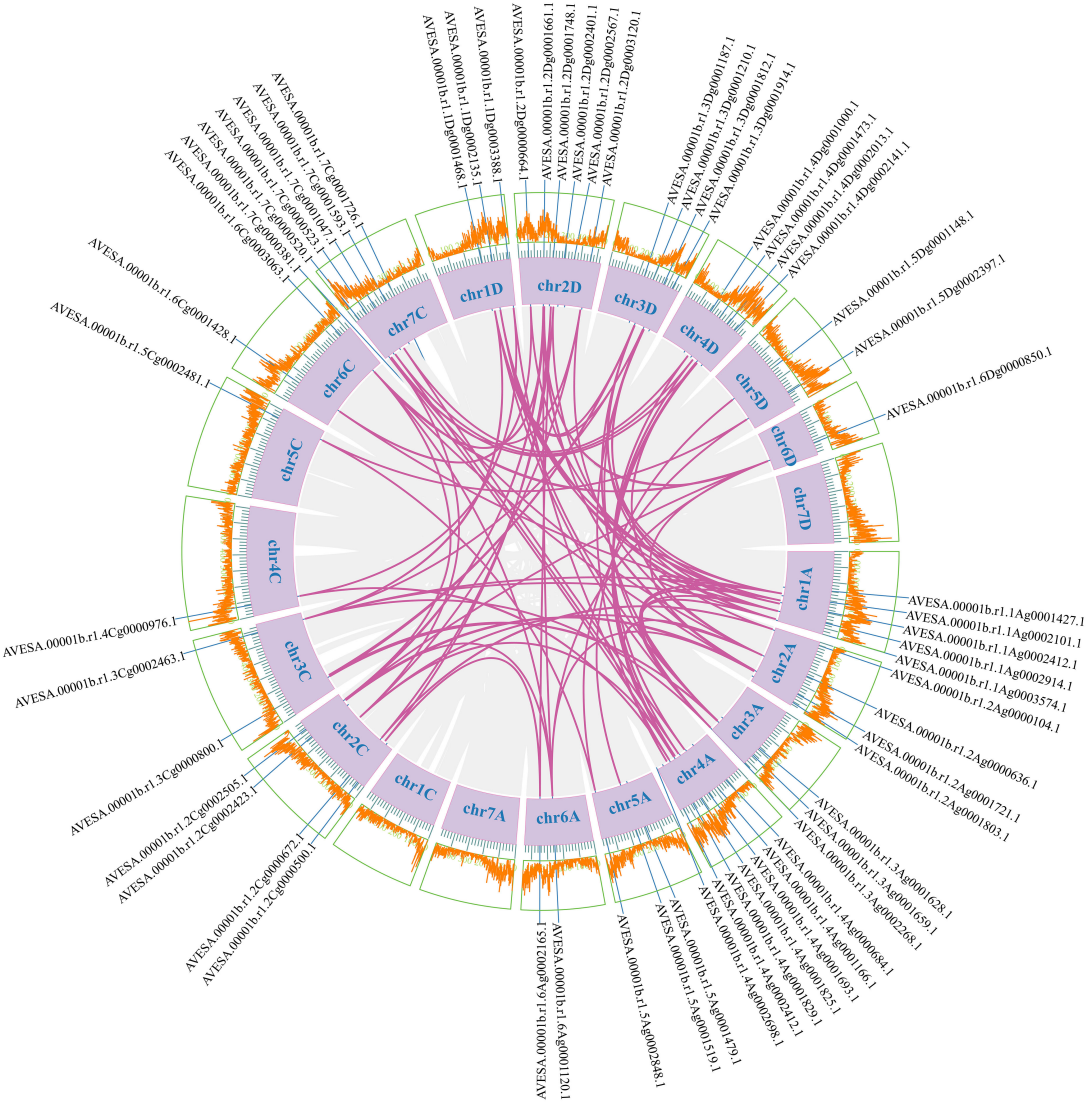
Collinear relationships of *AsCDPK* genes. The purple lines indicate duplicated *CDPK* gene pairs. The chromosome number is displayed next to each chromosome.

To investigate the evolutionary relationships of *CDPK* gene family members, we constructed a neighbor-joining (NJ) phylogenic tree using *CDPK* sequences from *Arabidopsis*, rice, and oat. A total of 123 CDPK members were classified into four groups (Group 1-4) (**Figure 1**; **Supplementary Table S3**), with AsCDPKs distributed across these groups. The chromosomal locations of *AsCDPKs* were identified using TBtools software, gene annotation data, and gene density analysis, revealing that all *AsCDPKs* were mapped to 18 chromosomes (**Figure 2**). Each of the 18 chromosomes contained 1-7 *AsCDPK* genes, with chromosomes chr4C, chr5C, and chr6D each only having one *AsCDPK* gene. In addition, collinearity analysis of *AsCDPKs* was conducted using MCScanX. The results revealed the presence of 78 pairs of segmental duplication genes among *AsCDPK* gene family, but no tandem duplication event (**Figure 3**). The selection pressure on gene pairs was evaluated by calculating the nonsynonymous substitution rate (*Ka*) and synonymous substitution rate (*Ks*), as well as the *Ka*/*Ks* ratio, for the identified paralogous *AsCDPK* gene pairs (**Supplementary Table S4**). The *Ka/Ks* value, an indicator for the selection history of these paralogous gene pairs, was below 0.300, indicating that these gene pairs underwent strong purifying selection during the evolutionary process, leading to the function of these gene pairs to be relatively conserved.

### 3.3 Structural analysis of *AsCDPK* genes

**Figure 4:**
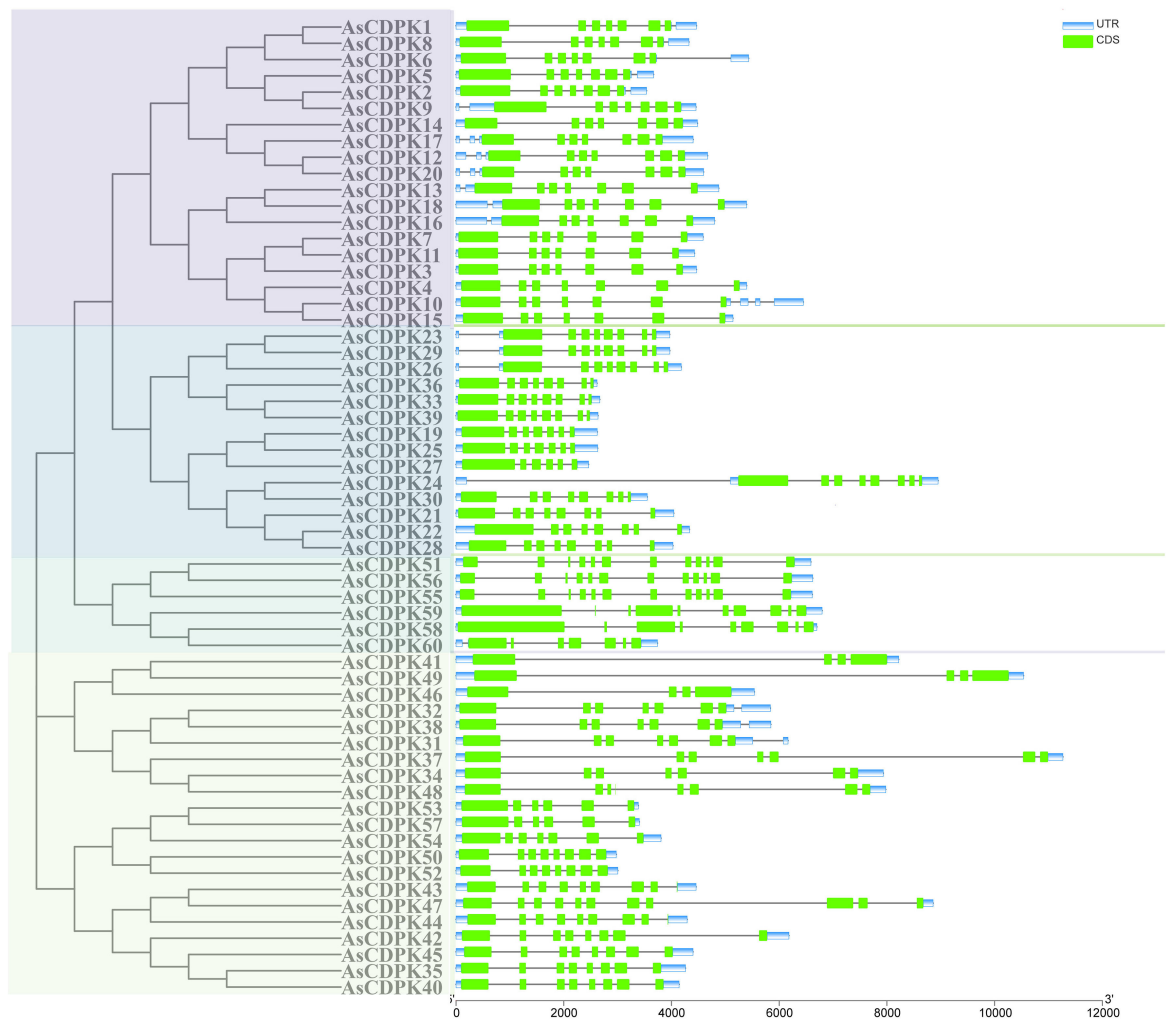
Gene structure analysis of *CDPK* gene members in oat. Green boxes represent exons, grey lines indicate introns, and blue boxes represent untranslated 5′and 3′ regions.

**Figure 5:**
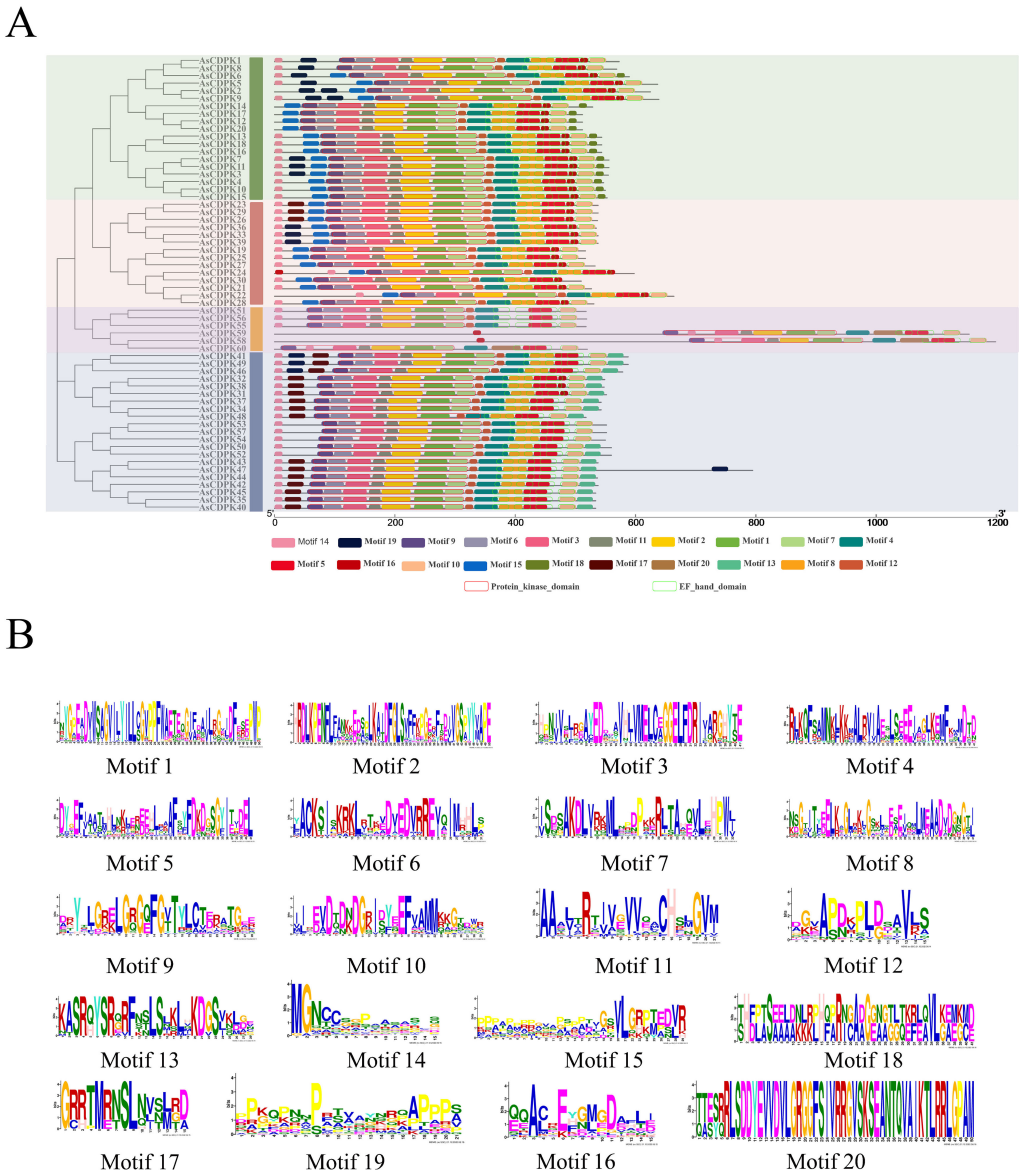
Protein structures of AsCDPKs in oat. **(A)** Protein structures of AsCDPKs. Different motifs are represented by specific colors. Red hollow box indicted the Serine/Threonine protein kinases domain (SM000220), and green hollow box indicted the EF-hand (SM000054). **(B)** Conserved motifs of *AsCDPKs* predicted by MEME.

Gene structure analysis provides insights into potential evolutionary relationships among gene families. To better understand the diversity of *AsCDPK* genes, we constructed an unrooted phylogenetic tree (**Figure 1**) using the full-length AsCDPK protein sequences and compared this with the gene structure of the corresponding gene sequences (**Figure 4**). The analysis of the evolutionary relationship aiming these *CDPKs* showed that most of the *AsCDPK* members within the same subfamily exhibited similar exon/intron structures. Conserved motifs in homologous proteins may serve functional roles. To further investigate the structural diversity of A*sCDPKs*, we conducted a comparative analysis of the unrooted phylogenetic tree, focusing on the conserved motifs and domain combinations with the respective gene sequences (**Table 1**; **Figure 5A**). Totally, 20 of the most conserved motifs were identified among oat *AsCDPKs* using online MEME tools (**Figure 5B**). The unrooted phylogenetic tree revealed that *AsCDPKs* could be divided into four subgroups. All AsCDPK members contained a protein kinase domain, which was composed of motifs 9, 6, 3, 11, 2, 1, and 7 as illustrated in **Figure 5**. Motif 9 and 2 were detected as ATP binding site and serine/threonine-protein kinase active site, respectively.

**Figure 6:**
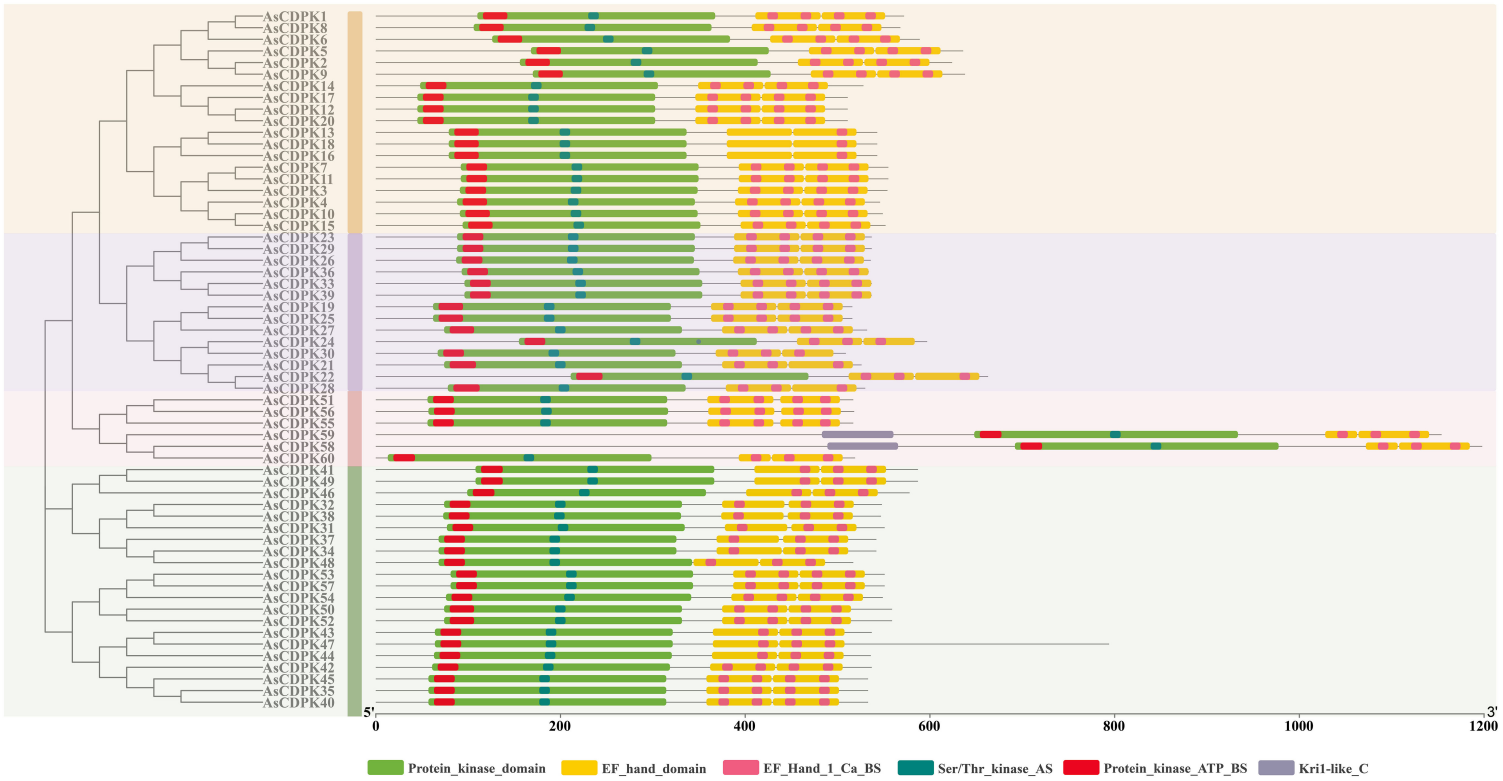
Conserved domain and important sites of AsCDPKs in oat. Different active sites are indicated by different colored boxes.

In oat, 55 CDPKs possessed four Ca^2+^ binding EF-hand motifs, composed of motifs 4, 8, 5, 16, 10, and 20. Furthermore, the five AsCDPKs (AsCDPK24/30/58/59/60) each contained three EF-hand structures (**Figure 5**). All AsCDPK members contained motifs 4, 8, 5 and 10, except for the third subfamily without motif 8. Despite variations in motif compositions among different subfamilies, CDPK proteins of the same type typically displayed similar motif components. All AsCDPK members possess ATP-binding sites and serine/threonine-protein kinase active sites within their kinase domains (**Figure 6**). 95% of AsCDPK members contained three or four Ca^2+^ binding sites in the EF-hand conserved region, but AsCDPK13/18/16 just contained one Ca^2+^ binding site. Additionally, AsCDPK59 and AsCDPK58 contained the Kri1-like_C site. In summary, the conserved motif structures within each subgroup supported a close evolutionary relationship between them. However, functional differences may exist among members of different subgroups.

### 3.4 *Cis*-acting elements in the promoters of *AsCDPK* genes

**Figure 7:**
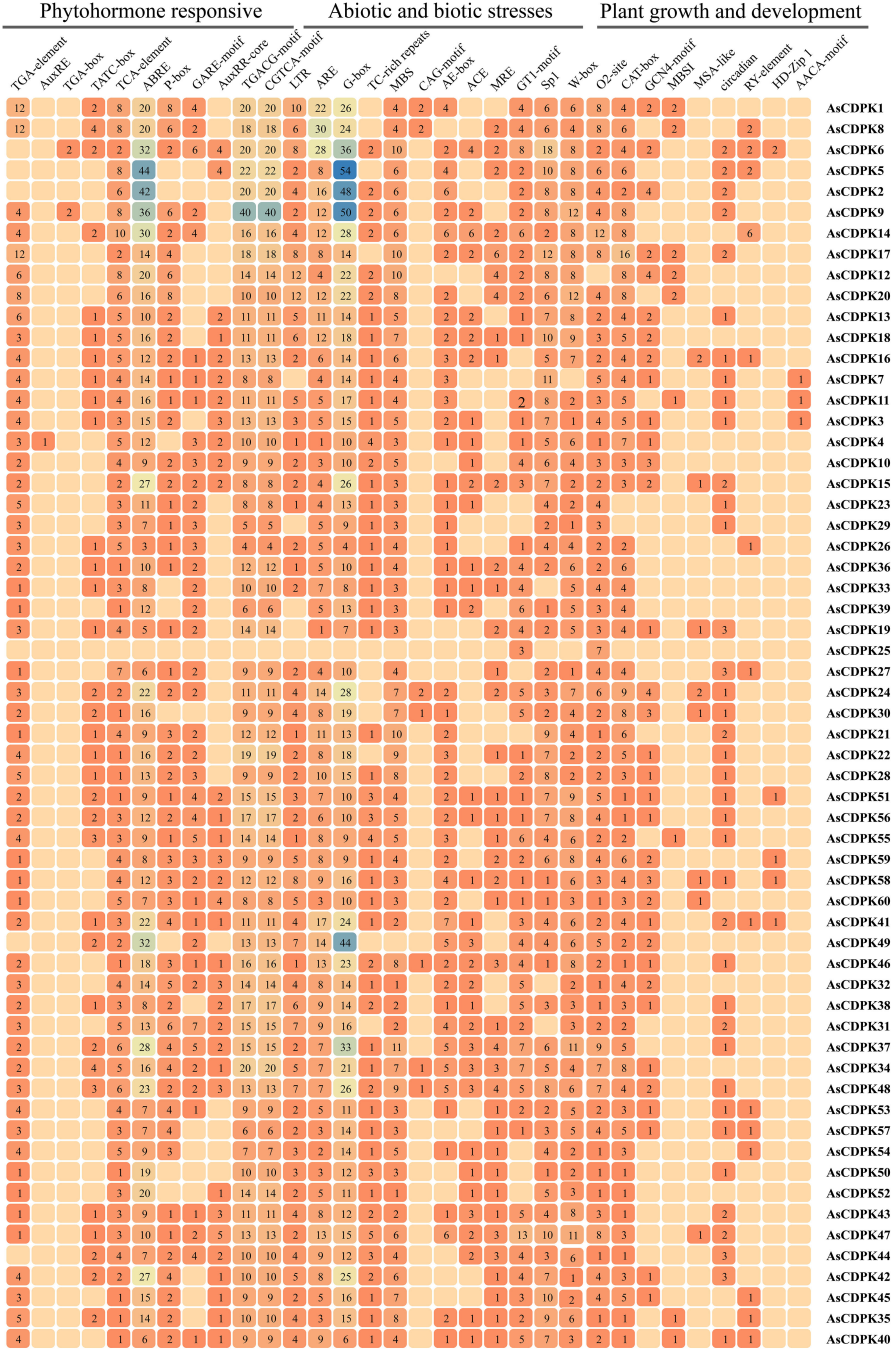
Analysis of *cis*-acting regulatory elements in the promoter regions of *AsCDPK* genes. The different colors and numbers of the grid indicate the numbers of different promoter elements in these *CDPK* genes.

To further elucidate the functions and regulatory mechanisms of *AsCDPK* genes in plant development and stress responses, we performed a cis-element analysis of the 2,000 bp promoter region upstream of the start codon for each *AsCDPK* gene using the PlantCare database. A variety of *cis*-acting regulatory elements were identified and classified into categories such as phytohormone response, biotic and abiotic stress, and plant growth and development (**Figure 7**). Transcription factor-associated *cis*-element (W-box, MBS, MRE and MBSI) were conserved in all *AsCDPK* genes. *AsCDPK* gene promoters generally contained *cis*-elements associated with phytohormone response and biotic and abiotic stress. However, *cis*-elements related to plant growth and development were only present in a few *AsCDPK* gene members. For the predicted *cis*-elements in the promoter, *AsCDPK25* just contained the GT1-motif and O2-site elements, while the other members contained the *cis*-elements associated with phytohormone response (TCA-element, ABRE, TGACG-motif, and CGTCA-motif) and biotic and abiotic stress (ARE and G-box). The auxin response *cis*-element (AuxRE or TGA-box) was only present in the promoters of *AsCDPK4*/*9*/*6* genes. For the predicted *cis*-elements related to plant growth and development, only *AsCDPK7*/*11*/*3* had endosperm-specific negative expression *cis*-elements (AACA-motif). Furthermore, *AsCDPK36/33/39/52* exclusively possessed regulatory *cis*-elements related to zein metabolism (O2-site) and meristem-expression element (CAT-box). Approximately 98% of *AsCDPKs* contained *cis*-elements responsive to phytohormone and stress, indicating that *AsCDPK* genes with these *cis*-elements might be responsive to stress and plant hormones.

### 3.5 Expression patterns of *AsCDPK* genes under different salt stresses

**Figure 8:**
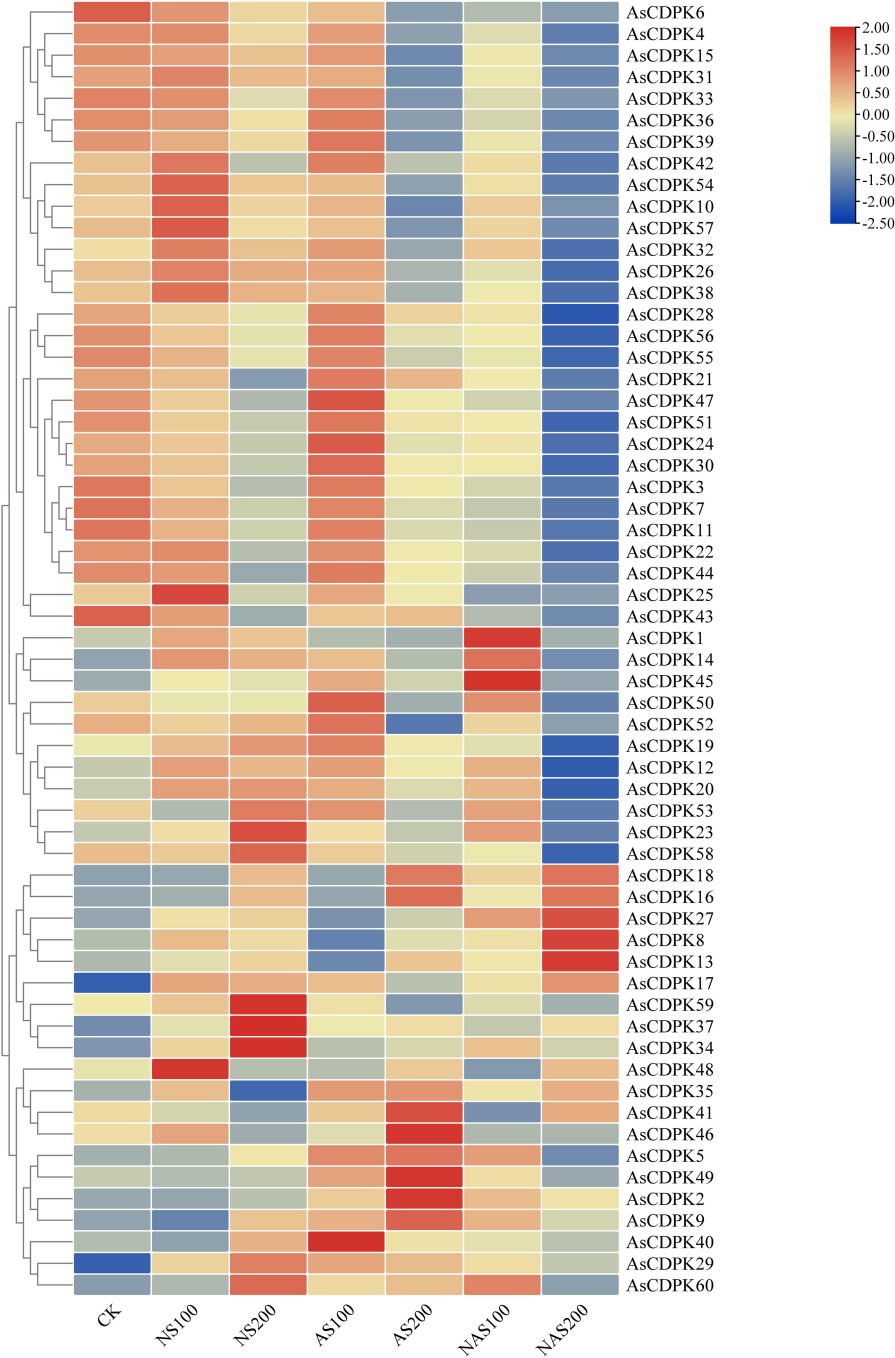
Expression analysis of *AsCDPK* genes in oat under different salt stresses. CK: normal treatment; NS100: 100 mmol·L^-1^ neutral salt; NS200: 200 mmol·L^-1^ neutral salt; AS100: 100 mmol·L^-1^ alkaline salt; AS200: 200 mmol·L^-1^ alkaline salt; NAS100: 100 mmol·L^-1^ mixed salt-alkali; NAS200: 200 mmol·L^-1^ mixed salt-alkali.

**Figure 9:**
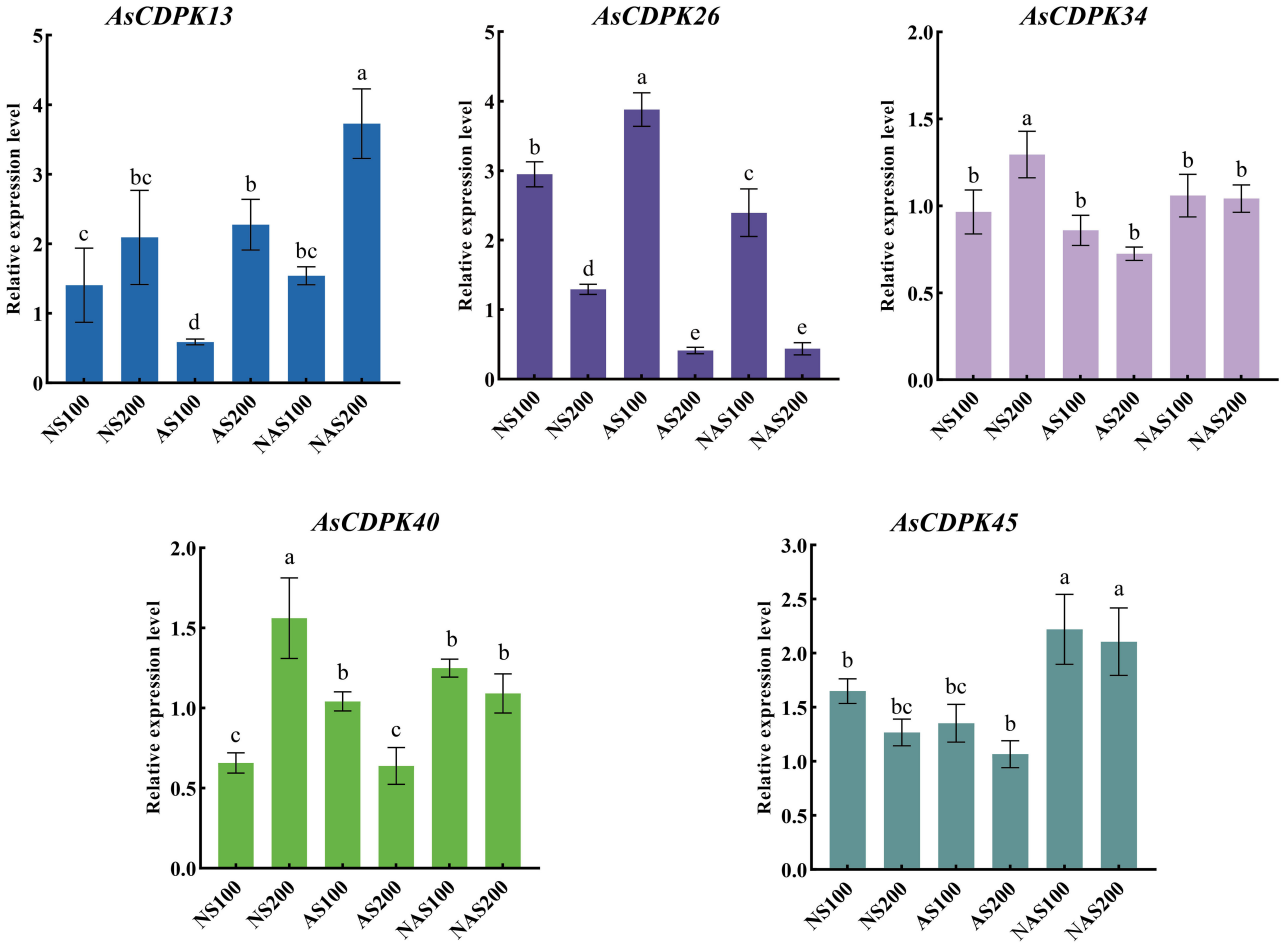
Relative expression levels of *AsCDPK* genes under different salt stresses. Different lowercase letters indicate significant difference at *P*<0.05.

Analysis of gene expression patterns could provide valuable insights into the biological functions of the genes. To explore the roles of the *AsCDPK* genes in response to salt stress, we examined their expression patterns under different salt stress doses (**Figure 8**; **Supplementary Table S5**). The results showed that all 59 *AsCDPK* genes, with the exception of *AsCDPK25*, were expressed under the seven treatments (CK, NS100, NS200, AS100, AS200, NAS100, and NAS200). Among these genes, *AsCDPK42/54/10/57/32/26/38* exhibited the similar expression patterns. Relative to normal growth conditions, these gene expression levels were upregulated with NS100 treatment and downregulated with AS200 and NAS200 treatments. Furthermore, the expression of these genes decreased as salt dosage increased. Although the number of up-regulated or down-regulated *AsCDPK* members varied across treatments, 75% of the members showed down-regulated expression under the NAS200 treatment. Cluster analysis further confirmed that the majority of *AsCDPK* genes displayed the similar expression patterns, despite varying degrees of upregulation or downregulation. Notably, certain *AsCDPK* gene members showed significant up-regulation or down-regulation in response to saline-alkali treatment, suggesting their roles in the oat’s response process to salt stress. To investigate the expression of *AsCDPK* genes under salt stress, RT-qPCR analysis was conducted on the five differentially expressed genes identified from the transcriptome in the shoots (**Figure 9**). The expression profiles of the five *AsCDPK* genes were found to align with the transcriptome data from salt-stress tissues. Variations in the expression of AsCDPK genes, including up-regulation and down-regulation, were observed under different salt treatment dosages. Specifically, *AsCDPK34* and *AsCDPK40* exhibited significant differences under NS200 treatment, whereas *AsCDPK13/26/40* showed significant differences under NAS100 treatment. Moreover, *AsCDPK13* and *AsCDPK45* expressions were significantly different under the mixed saline-alkali treatments compared to the normal treatment. These findings highlighted the significant differences in the expression of the *AsCDPK* genes under various stress conditions, suggesting differential functions of these *AsCDPKs* in response to diverse stresses.

### 3.6 Correlation between *AsCDPK* and ion transporter gene expression

**Figure 10:**
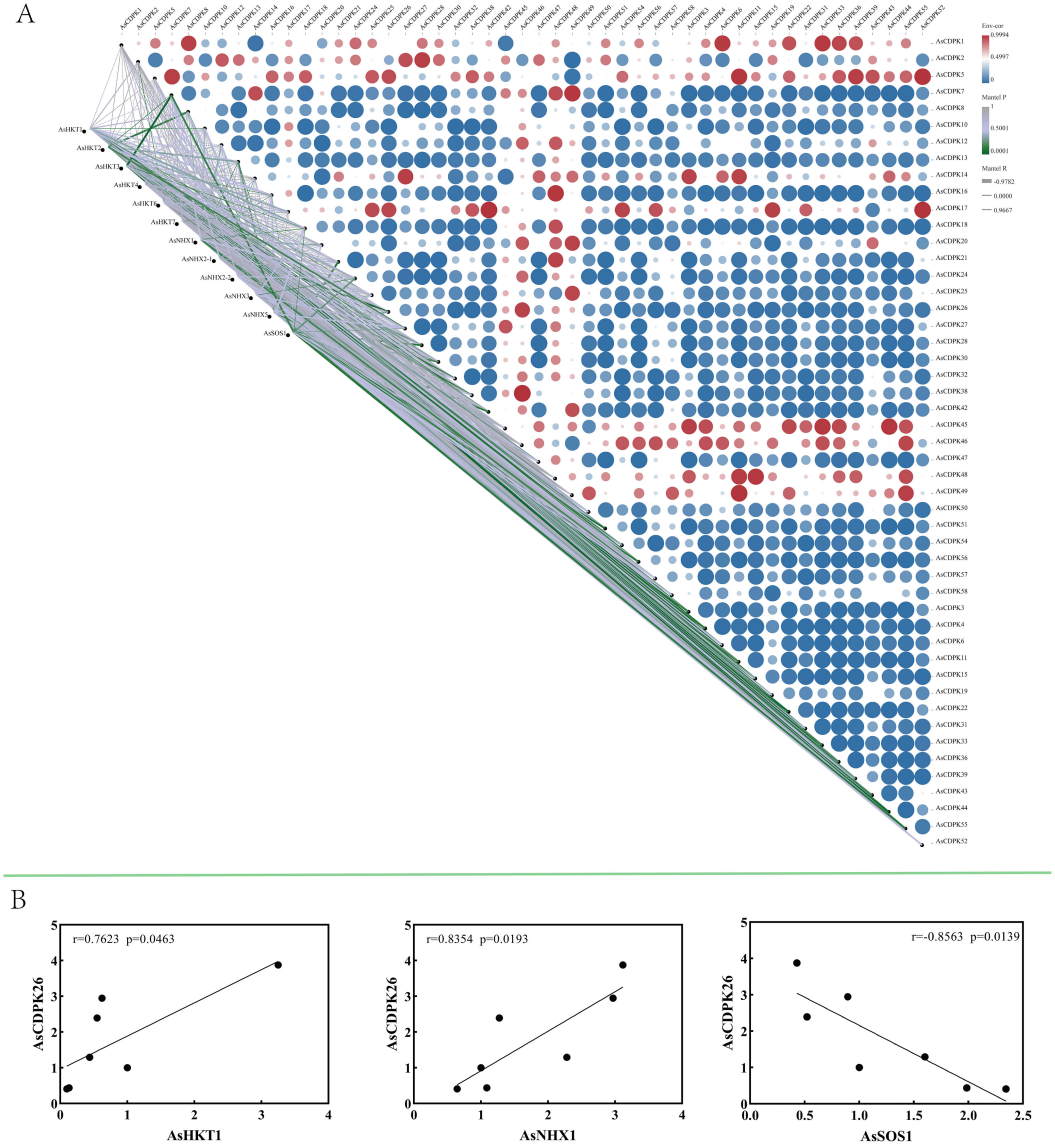
Expression correlation between *AsCDPKs* and genes related to ion transporters. **(A)** Correlation analysis was conducted between 49 significantly differentially expressed *AsCDPKs* and the expression of 12 ion transporter genes. **(B)** The correlation analysis between the expression levels of *AsCDPK26* and *AsHKT1*, *AsNHX1*, or *AsSOS1* genes were confirmed by RT-qPCR.

To investigate the role of *AsCDPK*’s in saline-alkali stress responses, *AsCDPKs* and the relevant ion transporters (*HKT1*, *HKT2*, *HKT3*, *HKT4*, *HKT6*, *HKT7*, *NHX1*, *NHX2-1*, *NHX2-2*, *NHX3*, *NHX5*, *SOS1*) were analyzed using oat transcriptome data. Gene expression correlation analysis showed a significant association between *AsCDPK* genes (excluding *AsCDPK5*) and ion transporters (*P <*0.05) (**Figure 10A**). Notably, 21 *AsCDPK* genes (*AsCDPK 2/3/4/6/7/10/11/13/21/22/26/28/33/36/39/42/44/49/51/55/56*) showed an extremely significant correlation (*P*<0.01) with the ion transporters including *AsHKT1*, *AsHKT2*, *AsHKT3*, *AsHKT6*, *AsNHX1*, *AsNHX5*, and *AsSOS1*. RT-qPCR analysis validated the correlation between *AsCDPK26* and *AsHKT1*, *AsSOS1*, and *AsNHX1* genes (**Figure 10B**). A positive correlation was found between *AsCDPK26* and both *AsHKT1* and *AsNHX1* genes, respectively (*p*<0.05), whereas a negative correlation was observed between *AsCDPK26* and the *AsSOS1* gene (*p*<0.05). These findings suggest that the function of *AsCDPK26* in response to salt stress may be modulated by the genes *AsHKT1*, *AsSOS1*, and *AsNHX1*.

### 3.7 Identification of the transformed algal strains expressing *AsCDPK26* gene

**Figure 11:**
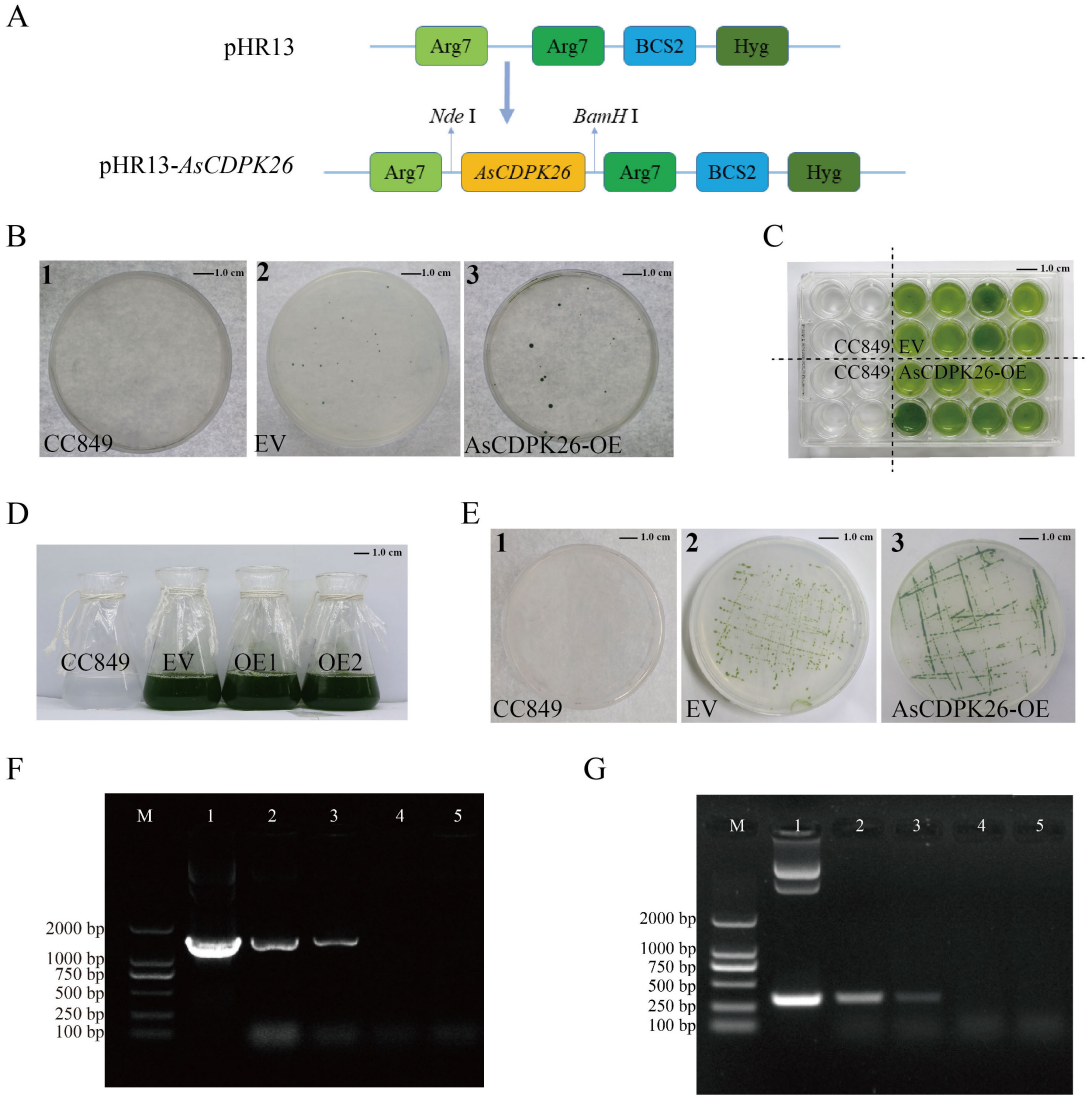
Screening and identification of transgenic algal lines of *AsCDPK26* gene. **(A)** Schematic diagram of expression vector. **(B)** Transgenic algal lines were screened by TAP agar plate containing 10 mg L^-1^ hygromycin. **(C)** 24-well screening plate for the transgenic lines. **(D)** Liquid culture of wild strain, empty vector (EV) and transformed strains (OE1 and OE2). **(E)** Subculture plate for the transformed lines. **(F)** DNA identification of pHR13-*AsCDPK26*-transgenic *C. reinhardtii.* M: DNA marker DL2000; 1: Positive control (plasmid); 2-3: Positive transgenic lines; 4: Negative control (wild strain CC849); 5: EV. **(G)** PCR analysis of pHR13-*AsCDPK26*-transgenic *C. reinhardtii*. M: DNA marker DL2000; 1: Positive control (plasmid); 2-3: RT-PCR identification of the transformed lines; 4: Negative control (wild strain CC849); 5: EV.

The expression analysis above (**Figure 8**) indicated that a set of *AsCDPK* genes expressed highly up on different types of salt stresses, including *AsCDPK26/34/37/42/2/5/13/16/18/45/21/28/30/40/47/51/55/56*. Notably, *AsCDPK26* gene was highly expressed under neutral salt, alkaline salt, and mixed salt-alkali stresses (**Figure 9**) revealed by RT-qPCR. Furthermore, AsCDPK26 was detected to be the most closed with AtCPK27, evidenced by sequence comparative analysis (conserved domain and phylogenetic tree) using AtCPKs and AsCDPKs (**Supplementary Figure S1**). AtCPK27 has been reported to mediate salt stress tolerance in *Arabidopsis* by regulating ion and ROS homeostasis (Zhao et al., 2015). It is possible that AsCDPK26 has a similar function to AtCDPK27 in the response to salt stress. Consequently, we selected *AsCDPK26* gene for functional analysis in this study. To investigate the function of *AsCDPK26* gene, we constructed a novel expression vector for its heterologous expression in the single-cell model organism *C. reinhardtii*. The constitutive expression vector pHR13-*AsCDPK26* contained *AsCDPK26* gene and a hygromycin resistance gene (*Hyg*) (**Figure 11A**). Wild-type *C. reinhardtii* CC849 could not grow on plates containing hygromycin (10 mg·L^-1^) (**Figure 11B**). Consequently, the transgenic strains and empty vector (EV) lines were selected on TAP solid plates containing 10 mg·L^-1^ of hygromycin, and the transgenic algal line was cultured in TAP liquid medium (containing hygromycin) for further experiments (**Figures 11C–E**). The electrophoresis results confirmed that a band matching the size of the plasmid DNA was successfully amplified in the positive transgenic strain, whereas no target band was detected in the negative control and wild type *C. reinhardtii* CC849. This suggested that the *AsCDPK26* gene had been integrated into the *C. reinhardtii* genome and remained stable genetically (**Figure 11F**). The total RNA of the wild type *C. reinhardtii* CC849, EV, and the transformed algal strain was separately extracted, followed by PCR analysis using the primers specific to the target gene. As expected, the specific fragment of *AsCDPK26* gene was not detected in the wild type or EV cells, but was successfully amplified in the transgenic strains (**Figure 11G**). This result demonstrated successful expression of the exogenous *AsCDPK26* gene in the algal cells.

### 3.8 Overexpression of *AsCDPK26* gene enhances salt tolerance of *C. reinhardtii*

**Figure 12:**
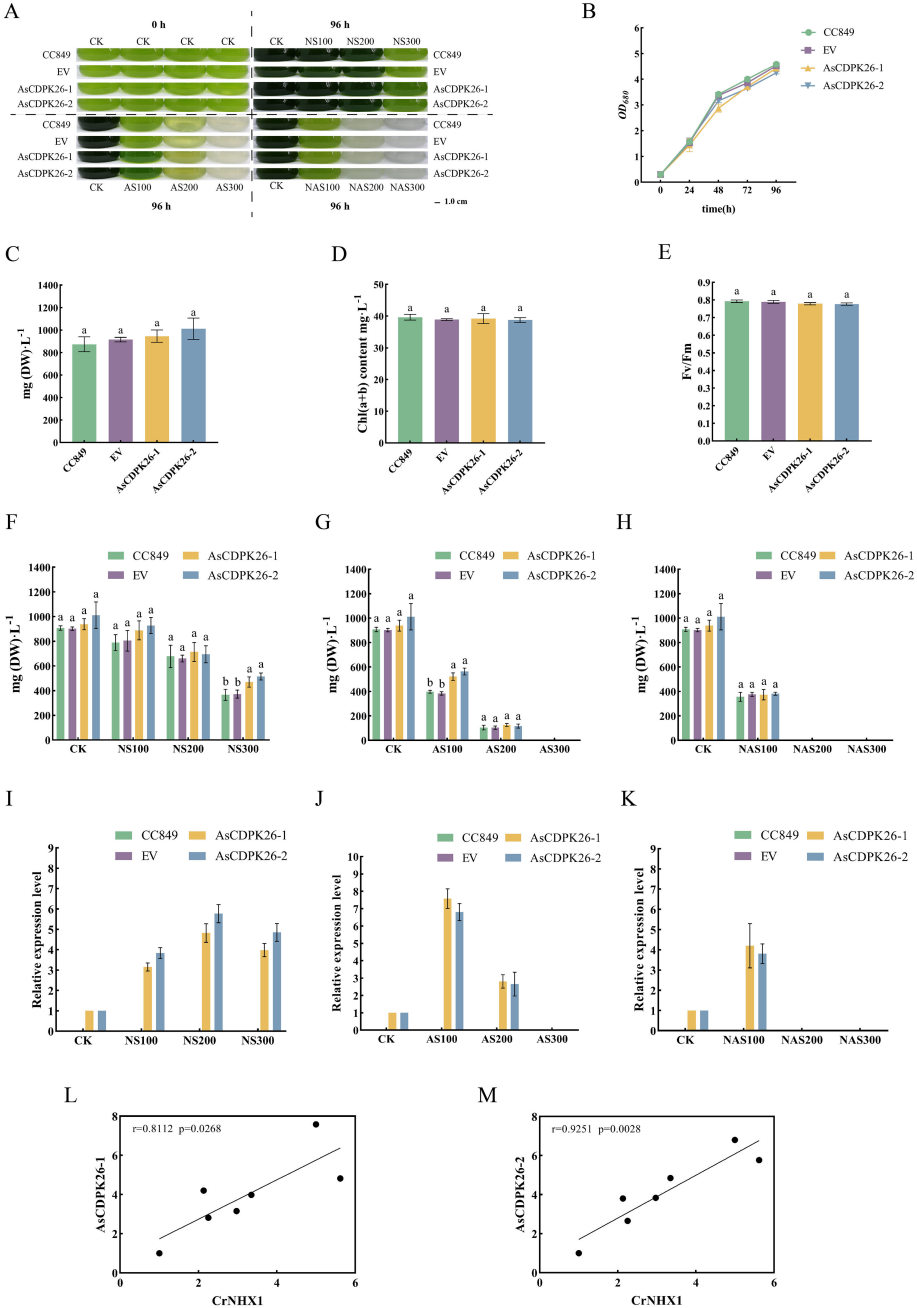
Effect of salt treatment on the algal growth of different genotypes. **(A)** Growth of *C. reinhardtii* CC849 and the transgenic algal strains exposed to different doses of salt stresses for 96 h **(B)** Growth curves of the wild type, EV and transformed algal strains under normal treatment. **(C)** The biomass, **(D)** total chlorophyll contents, and **(E)** Fv/Fm value of the wild type, EV and transgenic algal strains were measured after 96 hours of cultivation under normal conditions. **(F-H)** The biomass of wild type, EV and transformed algal strains were measured after 96 hours of cultivation under different doses of salt stresses. CK: normal treatment; NS100: 100 mmol·L^-1^ neutral salt; NS200: 200 mmol·L^-1^ neutral salt; NS300: 300 mmol·L^-1^ neutral salt; AS100: 100 mmol·L^-1^ alkaline salt; AS200: 200 mmol·L^-1^ alkaline salt; AS300: 300 mmol·L^-1^ alkaline salt; NAS100: 100 mmol·L^-1^ mixed salt-alkali; NAS200: 200 mmol·L^-1^ mixed salt-alkali; NAS300: 300 mmol·L^-1^ mixed salt-alkali. **(I-K)** The *AsCDPK26* gene expression level in the wild type, EV and transformed algal strains were measured after 96 hours of different salt treatments. **(L, M)** RT-qPCR verification was conducted to analyze the correlation between the expression levels of *AsCDPK26* and *NHX1* genes in *C. reinhardtii*. Different lowercase letters indicate significant difference at P<0.05.

To investigate the function of the exogenous *AsCDPK* genes in *C. reinhardtii*, a single-cell photosynthetic model plant, we first examined the growth of the wild type, EV and transgenic strains under normal culture conditions for comparison. Similar growth properties were observed for the wild type, EV and the transgenic strain under normal growth conditions (**Figures 12A, B**). No significant difference in dry weight at 96 hours (**Figure 12C**) and total chlorophyll contents (**Figure 12D**) were observed between the wild type and transgenic strains. Furthermore, the Fv/Fm values were consistent with the change in pigment content for the wild type, EV and transgenic strain (**Figure 12E**). These findings indicated that the overexpression of the exogenous *AsCDPK26* gene did not affect the growth and photosynthesis of the algal cells. After treatment with 100 mM and 200 mM neutral salt for 96 hours, no significant difference in dry weight was observed between the wild type and transgenic strains (**Figure 12F**). However, when the neutral salt concentration reached 300 mM, the dry weight of the transformed algal lines (*AsCDPK26-1* and *AsCDPK26-2*) was significantly increased by 1.29 and 1.41 times, respectively, compared to the wild strain (**Figure 12F**). Moreover, RT-qPCR analysis showed that the expression of *AsCDPK26* gene in *C. reinhardtii* was significantly upregulated under neutral salt treatment conditions (**Figure 12I**). After subjecting the algal cells to 96 hours of alkaline salt treatment at a 100 mM, the transformed strain showed improved growth compared to both the wild strain and the empty vector control (**Figure 12A**). Furthermore, the dry weight of the transformed strain (*AsCDPK26-1* and *AsCDPK26-2*) was significantly higher, with increases of 1.36 and 1.47 times, respectively, compared to the wild type strain (**Figure 12G**). Under AS200 treatment, no significant difference in dry weight were observed among the transformed strain, the wild type strain, and the empty vector strain (**Figure 12G**). However, under AS300 treatment, all algal strains died. The expression of the AsCDPK26 gene was observed to significantly increase under alkaline salt treatment (**Figure 12J**). Moreover, under high-dose mixed salt-alkali treatments (NAS200 and NAS300), none of the algal strains survived (**Figures 12A, H**). On the other hand, under low-dose mixed salt-alkali treatments (NAS100), the growth of the transformed strain showed no significant difference compared to that of the wild type strain and the empty vector strain (**Figure 12H**). However, the expression of the *AsCDPK26* gene in the transformed strain was significantly upregulated (**Figure 12K**). The expression profile of *AsCDPK26* in oat showed the similar patterns in *C. reinhardtii.* In *C. reinhardtii*, the gene expression of *AsCDPK26* increased under all salt treatments, with the most significant upregulation observed under low-dose alkaline salt treatment (AS100). This trend is consistent with the expression patterns of *AsCDPK26* in oat. However, under neutral salt treatment, the expression of *AsCDPK26* in *Chlamydomonas* initially increased and then decreased as the salt dose increased, although it remained higher than the control (CK).

To further elucidate the salt tolerance mechanism in the *AsCDPK26-*overexpressed algal strain, we performed RT-qPCR analysis to assess the correlation between *AsCDPK26* and the ion transporter gene (*NHX1*) under various salt treatments. The results demonstrated a positive correlation in gene expression levels (*p*<0.05) (**Figures 12L, M**), aligning with predictions made in oat. These collective findings suggested that overexpression of *AsCDPK26* might enhance salt tolerance in *C. reinhardtii* by interacting with the NHX1.

## 4 Discussion

### 4.1 Characteristics of *CDPK* genes in oat

*CDPK g*enes present in photosynthetic organisms play a crucial role in regulating various physiological processes, including hormone signaling and stress resistance. They are also critical for plant growth and development (Mehlmer et al., 2010; Boudsocq and Sheen, 2013). *CDPK* genes have been identified in different plant species, such as *A. thaliana* (Cheng et al., 2002), *O. sativa* (Asano et al., 2005), and *Solanum. lycopersicum* (Rutschmann et al., 2002). In this study, we identified 60 *AsCDPK* genes based on the oat genome database. By comparing them with *CDPK* members in *Arabidopsis* and rice, these *AsCDPKs* were classified into four groups (**Figure 1**), like the cases in *A. thaliana* and *Brachypodium distachyon* (Cheng et al., 2002; Wen et al., 2020). Phylogenetic analysis revealed that *CDPKs*, widely present in plants, are relatively conserved. Despite the exon-intron patterns and motifs of *AsCDPKs* being conserved and similar to those of *B. distachyon* (Wen et al., 2020), variations were observed among different subfamilies of AsCDPKs in the phylogenetic tree, suggesting that AsCDPKs may serve diverse functions (**Figure 4**). *AsCDPK*s primarily underwent segmental duplication, which might be the main driving force for the evolution of *AsCDPK* gene family. Furthermore, the uneven distribution of *AsCDPKs* across chromosomes might correlate with species evolution and genetic variation (**Figure 2**) (Miao et al., 2023). Notably, similar distributional patterns have been observed in other plant species (Miao et al., 2023).

All AsCDPKs identified in our study exhibited the typical characteristic structures of CDPK family, including a variable N-terminal domain, a catalytic Ser/Thr protein kinase domain, an autoinhibitory domain, and an EF-hand domain. Members in the same subfamily exhibited the similar exon-intron structures, biochemical properties, and conserved motif compositions, indicating a close evolutionary relationship among them. The protein kinase domain catalyzes the transfer of the γ-phosphate from ATP to specific amino acids in proteins, resulting in conformational changes and functional alterations (Hanks et al., 1988). AsCDPKs are a type of protein kinase that contain ATP-binding sites (**Figure 6**). CDPK use ATP as a source of phosphate groups to regulate target substrates in signal transduction pathways through phosphorylation (Wen et al., 2020). The calmodulin-like domain, featuring an EF-hand structure, binds Ca^2+^, allowing CDPKs to act as Ca^2+^ sensors. Except for *AsCDPK24/30/58/59/60*, all *AsCDPKs* contain four EF-hand structures (**Figure 5**). This variation in the number of EF-hand structures was also observed in *CDPKs* from *A. thaliana*, *O. sativa*, and *Z. mays* (Cheng et al., 2002; Asano et al., 2005; Kong et al., 2013). EF hands of CDPKs act as a calcium sensor in calcium-binding affinities (Hu et al., 2016). In agreement with this, AtCPK25 was calcium independent owing to lacking of the functional EF hands (Boudsocq and Sheen, 2013). Therefore, these CDPKs like AsCDPK24/30/58/59/60 might be insensitive to the changes in cellular calcium in plants. Myristoylation is a lipid modification commonly observed in proteins, which enhances their interaction with cell membranes and contributes to protein localization and functional regulation (Wen et al., 2020). Among AsCDPK members, only AsCDPK2/14/22/24/58/59/60 lacked N-myristoylation sequences (**Table 1**). Furthermore, subcellular localization prediction indicated that AsCDPK proteins were typically located in chloroplasts, cytoplasm, and mitochondria. Previous studies have shown that CDPK has both N-myristoylation and palmitoylation sites, which are crucial for determining subcellular localization (Zhao et al., 2021a). For instance, MtCDPK4/14/16/22 and MtCRK6, which contain both N-myristoylation and palmitoylation sites, are localized to the plasma membrane. MtCDPK7/9/15 without N-acylation site or just having one site are distributed in the cytoplasm and nucleus (Zhao et al., 2021a). Thus, it is likely that the subcellular localization of CDPKs is influenced by other factors that require further investigation.

### 4.2 AsCDPKs may function importantly in multiple life processes, especially in stress responses

The analysis of *cis*-acting elements in gene promoter and gene expression patterns can provide valuable insights into the potential functions of the interested genes. Multiple *cis*-acting elements responsible for phytohormones, stresses, growth and development were observed in the promoter regions of *AsCDPKs*, indicating the potential role of *AsCDPKs* in regulating multiple responses to phytohormones, environmental stresses, and development (**Figure 7**). An increasing evidence has consistently identified that *CDPKs* play an important role in a variety of abiotic stresses, including drought, salinity, cold, nutrient deficiency, light, hypotonic stress, and so on (Harmon et al., 2001). *MtCDPK22* transcript is specifically and strongly expressed under cold stress, and its homologous gene *OsCPK17* is involved in cold stress response (Zhao et al., 2021a). *SlCDPK5/6*, *SlCDPK22/27* were highly induced under high temperature in tomato (Hu et al., 2016). Notably, GT1 and W-box elements detected in the *AsCDPK* promoters were considered to be the *cis*-acting elements in response to salt stress (Park et al., 2004; Zhou et al., 2008). The *cis*-acting GT-1 element was also identified to plays a key role in mediating the function of *SCaM-4* in inducing salt stress through interaction with GT-3b, a GT-1-like transcription factor (Park et al., 2004). *OsWRKY54* physically bound to the promoter sequences of *OsHKT1;5* via its W-box motif, promoting expression of the target gene (Huang et al., 2022). These *AsCDPK* genes (e.g. *AsCDPK10/26/34/37* and so on) contain dehydration-responsive elements (GT1 or W-box), were upregulated under neutral salt treatment (**Figure 7**; **Figure 8**). In *Arabidopsis*, some *AtCPKs* are involved in regulating phytohormone and abiotic stresses signaling when specific cis-acting elements were detected in the promoter regions (Zhang et al., 2020; Li et al., 2016; Huang et al., 2018). Thus, an analysis of similarities and differences between family members based on their expression pattern and promoter can provide candidate genes for further functional analysis at least in part.

### 4.3 *AsCDPK* gene exhibited specific expression patterns under various salt stresses

In response to salt stress, plants can resist damage by maintaining balance of ion, osmotic potential, and reactive oxygen species (ROS) (Zhao et al., 2020). CDPK regulate the activity of transcription factors, ion channels, transporters, and other proteins involved in signaling pathways through phosphorylation, thereby controlling gene expression, ion balance, and ROS homeostasis (Yang et al., 2021). For example, in *Arabidopsis*, AtCPK3 can phosphorylate the vacuolar potassium channel TPK1 to regulate intracellular K^+^/Na^+^ balance in response to salt stress (Latz et al., 2013). Conversely, overexpression of *CPK23* renders plants more sensitive to drought and salt stress, whereas the T-DNA insertion mutant of *AtCPK23*, *cpk23*, exhibits an increased tolerance to these stresses compared to the wild type. This process may negatively regulate drought and salt resistance by inhibiting K^+^ uptake (Ma and Wu, 2007). Previous studies indicated that CDPKs may enhance plant tolerance to natural salt stress. However, the role of CDPKs in plant responses to alkaline salt and mixed salt-alkali stresses remains unclear. In this study, we identified the specific expression patterns of *AsCDPKs* under different salt treatments (**Figure 8**). The expressions of certain *AsCDPK* genes (*AsCDPK17/29/34/37*) were found to be enhanced under all salt treatments, indicating their potential regulatory functions. Similar gene-specific expression patterns have also been observed in *Prunus mume* and *B. distachyons* (Miao et al., 2023; Wen et al., 2020). The expression level of *CsCDPK6* was significantly up-regulation under NaCl treatment of cucumber seedlings (Zhu et al., 2021). Similarly, the gene expression of *AsCDPK10/25/26/32/38/42/48/54/57* in oat was also increased under NS100 stress (**Figure 8**). After 48 hours of neutral salt stress, *CgCDPK* expression decreased initially before increasing with the rise in NaCl dosage (Wang et al., 2017b). More likely, the gene expression of *AsCDPK1/8/10/14/19/25/26/32/38/42/46/48/54/57* in oat was also increased while *AsCDPK16/18/23/29/34/37/58/59/60* genes showed an increased expression under higher dosage of NaCl stress. This observation could be attributed to the variations in sequences and functional diversity present among *CDPK* members (**Figure 8**). Although the role of *CDPKs* in neutral salt stress has been extensively studied, the literature still lacks information on their role in alkaline salt and mixed salt-alkali stresses. Salinity stress is a widespread environmental problem (Zhou et al., 2024). Despite considerable efforts to address this issue, two crucial aspects have been neglected, i.e. salt–alkali stress and complex salt stress (Shi and Sheng, 2005). In fact, soil salinization from NaHCO_3_ and Na_2_CO_3_ may be more severe than that caused by neutral salts like NaCl and Na_2_SO_4_ (Shi and Sheng, 2005). In this study, as alkalinity levels increased, oat plants suffered greater damage, which corresponded to decreased expression levels of most *AsCDPKs*. Interestingly, a small subset of *AsCDPK* genes actively responded to these stresses, showing a significant increase in expression levels. Specifically, *AsCDPK23/58/59/37/34* genes were involved in response to neutral salt stress while *AsCDPK18/16/41/46/49/2* genes responded to alkaline salt stress. In contrast, *AsCDPK18/16/27/8/13* genes strongly responded to mixed salt-alkali stresses (**Figure 8**). RT-qPCR analysis further validated the expression pattern of *AsCDPK13/26/34/40/45* genes, indicating their potential role in mitigating plant stress damage and enhancing plant salt tolerance (**Figure 9**).

In addition, the *AsCDPK26* gene was found to be positively correlated with the transcription levels of *AsHKT1* and *AsNHX1* genes (*p*<0.05), but negatively correlated with that of *AsSOS1* (*p*<0.05) (**Figure 10**). Biotic and abiotic stresses, as well as certain intracellular stimuli, can alter cytosolic Ca^2+^ concentrations. As a calcium ion sensor, CDPK can recognize calcium signals and act directly or indirectly on downstream interacting proteins, thereby triggering a series of physiological responses (Pirayesh et al., 2021). Previous studies have demonstrated that exposure to salt stress leads to an elevation in the concentration of calcium ions within the cell, which is detected by the calcineurin B-like protein CBL4 (also known as SOS3). This protein subsequently interacts with the CBL-interacting protein kinase CIPK24 (SOS2). The resulting SOS3/SOS2 complex then localizes to the plasma membrane and triggers the activation of the membrane-bound Na^+^/H^+^ antiporter (SOS1) via phosphorylation (Qiu et al., 2003; Shi et al., 2002). In *Gossypium barbadense*, the protein-protein prediction study revealed that GbNHX7 is involved in the CBL-CIPK protein interaction pathway (Akram et al., 2020). The HKT gene in barley is regulated by Ca^2+^ signals. Specifically, HvCaM1 plays a crucial role in regulating Na^+^ transport by preferentially controlling the transcription of HvHKT1s in response to salt stress. This regulatory process leads to the downregulation of *HvHKT1;5* and the upregulation of *HvHKT1;1* through the interaction between HvCaM1 and CAMTA4 (calmodulin-binding transcription activator). Consequently, the Na^+^ transport in the roots is effectively regulated, enabling barley to achieve higher salt tolerance (Shen et al., 2020). Additionally, CaM protein can also regulate the expression of *AtHKT1;1*, *MtHKT1;1*, and *MtHKT1;2* (Galon et al., 2010; Shkolnik et al., 2019; Zhang et al., 2019). Thereby preventing the transportation of Na^+^ is not disrupted by external stress. Thus, it is hypothesized that in response to salt stress, AsCDPK26 may interact directly or indirectly with AsHKT1, AsNHX1, and AsSOS1 proteins to regulate Na^+^ transport, thereby reducing the damage from ion toxicity in oat (**Figure 10**).

### 4.4 AsCDPK26 may be the valuable target gene in biotechnology to enhance host tolerance to salt stress

To investigate the function of the *CDPK* gene, we performed an overexpression experiment of the *AsCDPK26* gene in *C. reinhardtii* CC849, a unicellular model plant. The algae strains were treated with three types of salt stress, and subsequently, the growth features of the transgenic algal strains were measured. Compared to other plants, *C. reinhardtii* grows quickly and requires minimal cultivation costs. Especially, it is easily genetically modified and is referred as the “green yeast” (Rochaix, 1995). Thus, this algal species has been widely used to study various metabolic regulations in plants, including photosynthesis, storage substance biosynthesis and stress responses. For example, the overexpression of *LHCSR* and *PsbS* resulted in the improvement of light tolerance in *C. reinhardtii* (Wilson et al., 2023). Additionally, the overexpression of native *ORANGE* (*OR*) gene and *OR* mutant in *C. reinhardtii* enhanced carotenoid and ABA accumulation, leading to the increased resistance to abiotic stresses (Yazdani et al., 2021). Another study showed that the overexpression of phosphoribosyl pyrophosphate synthase promotes the resistance of *Chlamydomonas* to ionizing radiation (Jung et al., 2021).

**Figure 13:**
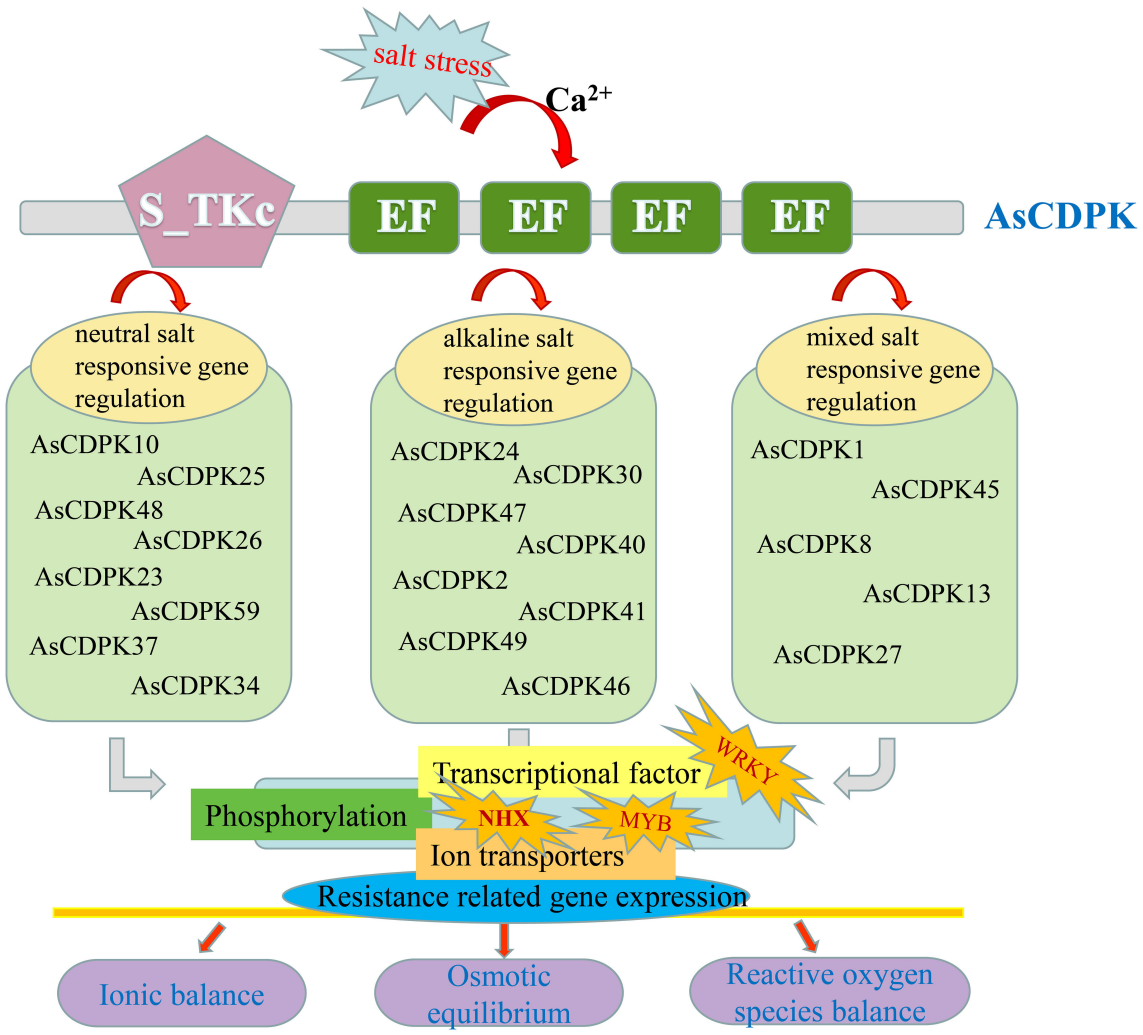
Possible mechanism of AsCDPKs-mediated responses to saline-alkali stresses in oat.

In this study, overexpression of *AsCDPK26* gene improved *C. reinhardtii* tolerance to various types of salt stresses. For example, overexpressing *AsCDPK26* improved the growth of *Chlamydomonas* in response to high-dose neutral salt treatment (**Figure 12**). Additionally, AsCDPK26 overexpression increased *Chlamydomonas*’ survival rate under AS100 treatment. *AsCDPK26* gene expression was significantly up-regulation, indicating that the transgenic cells have a stronger salt tolerance (**Figures 12I-K**). However, the algal strains did not survive when subjected to AS300, NAS200, and NAS300 treatments, respectively. Possibly, alkaline salt and mixed saline-alkali are more detrimental to plants than neutral salts (Shi and Sheng, 2005). In addition, *OsCPK4* overexpression plants accumulate less Na^+^ in their roots compared with control plants (Campo et al., 2014). The upregulation of ion transporter genes in roots of *OsCPK4* rice plants could be, at least in part, responsible for the observed lower level of Na^+^ accumulation in roots of *OsCPK4* rice plants (Campo et al., 2014). In our study, overexpressing the *AsCDPK26* gene in *C. reinhardtii* showed a significant correlation with the expression of the *NHX1* gene (*p*<0.05) (**Figures 12L, M**). This suggests that NHX1 plays an important role in improving the salt tolerance of *Chlamydomonas* by overexpressing the *AsCDPK26* gene. Previous studies have shown that CDPK in plants plays an important role in responses to biotic or abiotic stresses by phosphorylating specific substrate proteins or transcription factors (Yang et al., 2021; Latz et al., 2013; Zou et al., 2015; Atif et al., 2019). Therefore, we hypothesize that AsCDPK26 might interact with the ion transporter protein (NHX1) to regulate Na^+^ balance, thus enhancing salt tolerance in the transgenic strains. However, this hypothesis needed to be tested by yeast two-hybrid system. In summary, we hypothesized that a set of *AsCDPK* members might play key roles in oat response to salt stress by regulating the activity of a specific substrate protein (ion transporter) or transcription factor through phosphorylation (**Figure 13**). However, further experimental validation is necessary to elucidate the specific molecular mechanisms underlying this response.

## 5 Conclusions

In this study, a total of 60 *AsCDPK* gene members were identified in oat and classified into four subfamilies, followed by comprehensively clarifying their molecular characteristics, including gene structures, chromosomal locations, phylogenetic relationships, motif composition, domain distribution, synthesis analysis and *cis*-acting elements. Moreover, transcriptome analysis plus PCR verification revealed that a set of *AsCDPKs* (e.g. *AsCDPK13/26/34/40/45* and so on) might function importantly in responses to different salt stresses. The 49 differentially expressed *AsCDPKs* showed a significant positive correlation with the transcription levels of ion transporter genes *NHX* and *HKT* (*p*<0.05), yet a negative correlation with the transcription levels of the *SOS1* gene (*p*<0.05). This suggests that AsCDPKs may interact directly or indirectly with the ion transporters NHX, HKT, or SOS1, mediating the response of oats to salt stress. Importantly, overexpression of *AsCDPK26* gene in *C. reinhardtii* enhanced the host salt tolerance, highlighting the crucial role of *AsCDPK26* in the salt-stress signaling pathway. Further correlation analysis demonstrated a positive correlation between *AsCDPK26* and the transcription level of *NHX1* gene (*p*<0.05) in the transgenic *Chlamydomonas*. These results not only provide the first knowledge of function and structure of *AsCDPK* members in oat, particularly in responses to different salt stresses, but also establish a foundation for further exploration of the molecular mechanism underlying the abiotic stress responses in oat and other crops despite diverse functions of AsCDPKs needed to be proved experimentally in future.

## Data Availability

The datasets presented in this study can be found in online repositories. The names of the repository/repositories and accession number(s) can be found in the article/**Supplementary Material**.

## Supplementary material

Supplementary Figure 1 Phylogenic tree of AsCDPKs and AtCPK27.
