# Correction: Identification and expression analysis of calcium-dependent protein kinase family in oat (*Avena sativa* L.) and their functions in response to saline-alkali stresses

**DOI:** 10.3389/fpls.2026.1927897

**Published:** 2026-08-24

**Authors:** Ya-nan Li, Chunyan Lei, Qian Yang, Xiao Yu, Siming Li, Yan Sun, Chunli Ji, Chunhui Zhang, Jin-ai Xue, Hongli Cui, Runzhi Li

**Affiliations:** 1 College of Agriculture, Institute of Molecular Agriculture and Bioenergy, Shanxi Agricultural University, Jinzhong, Shanxi, China; 2 Key Laboratory of Coastal Biology and Bio-Resource Utilization, Yantai Institute of Coastal Zone Research, Chinese Academy of Sciences, Yantai, Shandong, China

**Keywords:** oat (*Avena sativa* L.), saline-alkali stresses, calcium-dependent protein kinase (CDPK*)*, *Chlamydomonas reinhardtii*, genetic transformation, Na+/H+ antiporter 1 (NHX1)

There was a mistake in **Figure 12A** as published. For the upper left corner figure of **Figure 12A**, the label for each row was erroneously labeled as the label for each column, respectively. The correct label of the upper left corner figure of **Figure 12A** is that “CC849, EV, AsCDPK26–1 and AsCDPK26–2” are labeled to the column “from left to right”, respectively, while “CK1, CK2, CK3 and CK4” are labeled to the row “from top to bottom”, respectively. The corrected **Figure 12** appears below.

**Figure 12:**
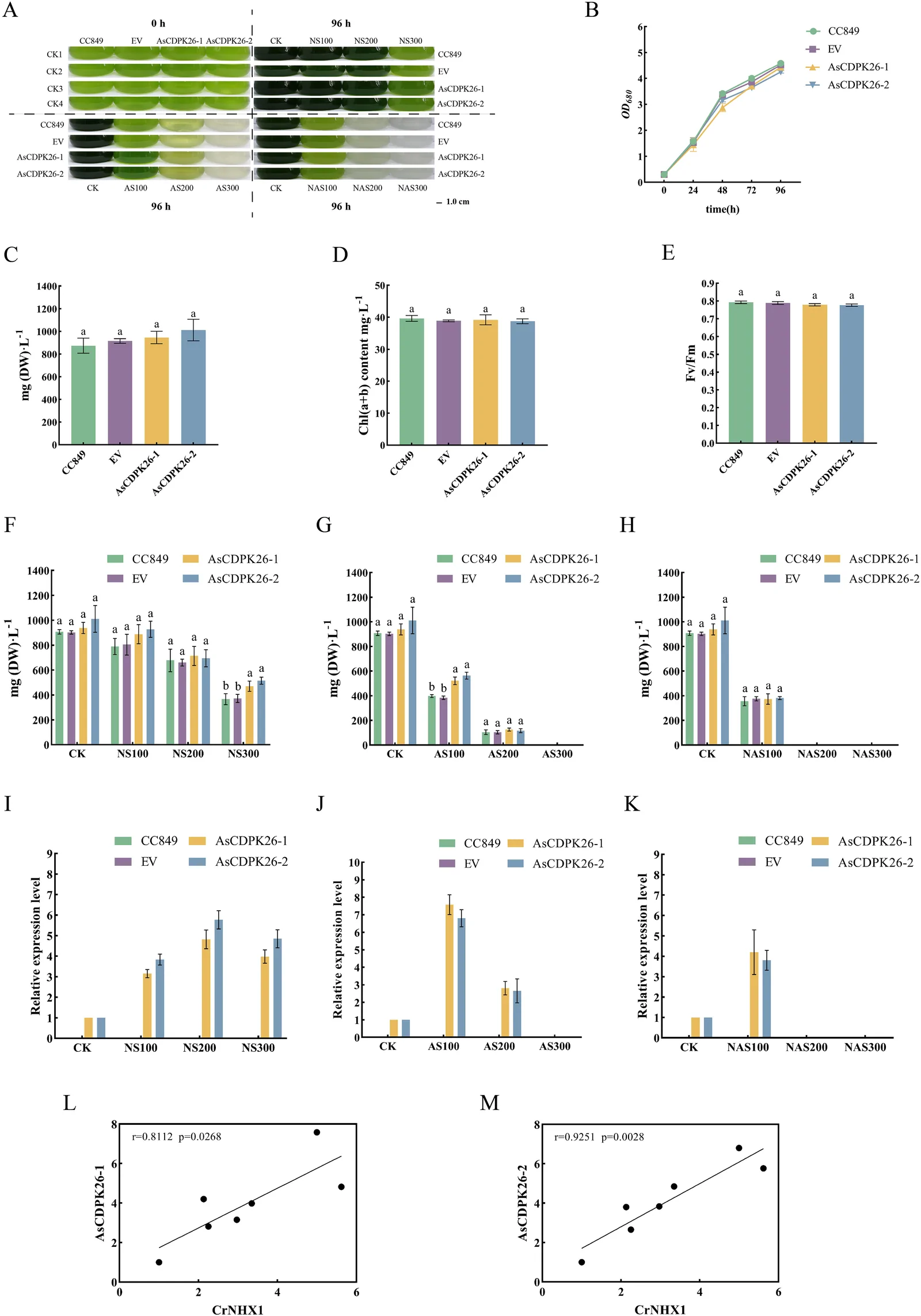
Effect of salt treatment on the algal growth of different genotypes. **(A)** Growth of *C. reinhardtii* CC849 and the transgenic algal strains exposed to different doses of salt stresses for 0 h and 96 h **(B)** Growth curves of the wild type, EV and transformed algal strains under normal treatment. **(C)** The biomass, **(D)** total chlorophyll contents, and **(E)** Fv/Fm value of the wild type, EV and transgenic algal strains were measured after 96 hours of cultivation under normal conditions. **(F–H)** The biomass of wild type, EV and transformed algal strains were measured after 96 hours of cultivation under different doses of salt stresses. CK: normal treatment; NS100: 100 mmol·L^-1^ neutral salt; NS200: 200 mmol·L^-1^ neutral salt; NS300: 300 mmol·L^-1^ neutral salt; AS100: 100 mmol·L^-1^ alkaline salt; AS200: 200 mmol·L^-1^ alkaline salt; AS300: 300 mmol·L^-1^ alkaline salt; NAS100: 100 mmol·L^-1^ mixed salt-alkali; NAS200: 200 mmol·L^-1^ mixed salt-alkali; NAS300: 300 mmol·L^-1^ mixed salt-alkali. **(I–K)** The *AsCDPK26* gene expression level in the wild type, EV and transformed algal strains were measured after 96 hours of different salt treatments. **(L, M)** RT-qPCR verification was conducted to analyze the correlation between the expression levels of *AsCDPK26* and *NHX1* genes in *C. reinhardtii*. Different lowercase letters indicate significant difference at *P*<0.05.

There was a mistake in the caption of **Figure 12** as published. The time for salt stresses was erroneously typed as “96 h” only, and “0 h” was missed. The corrected caption of **Figure 12** appears below.

The original version of this article has been updated.

