# Supplementary figures and images for: Identification and expression analysis of calcium-dependent protein kinase family in oat (Avena sativa L.) and their functions in response to saline-alkali stresses
Source: Front Plant Sci. 2024 Oct 10;15:1395696. doi: 10.3389/fpls.2024.1395696 (PMC11499199; doi:10.3389/fpls.2024.1395696)

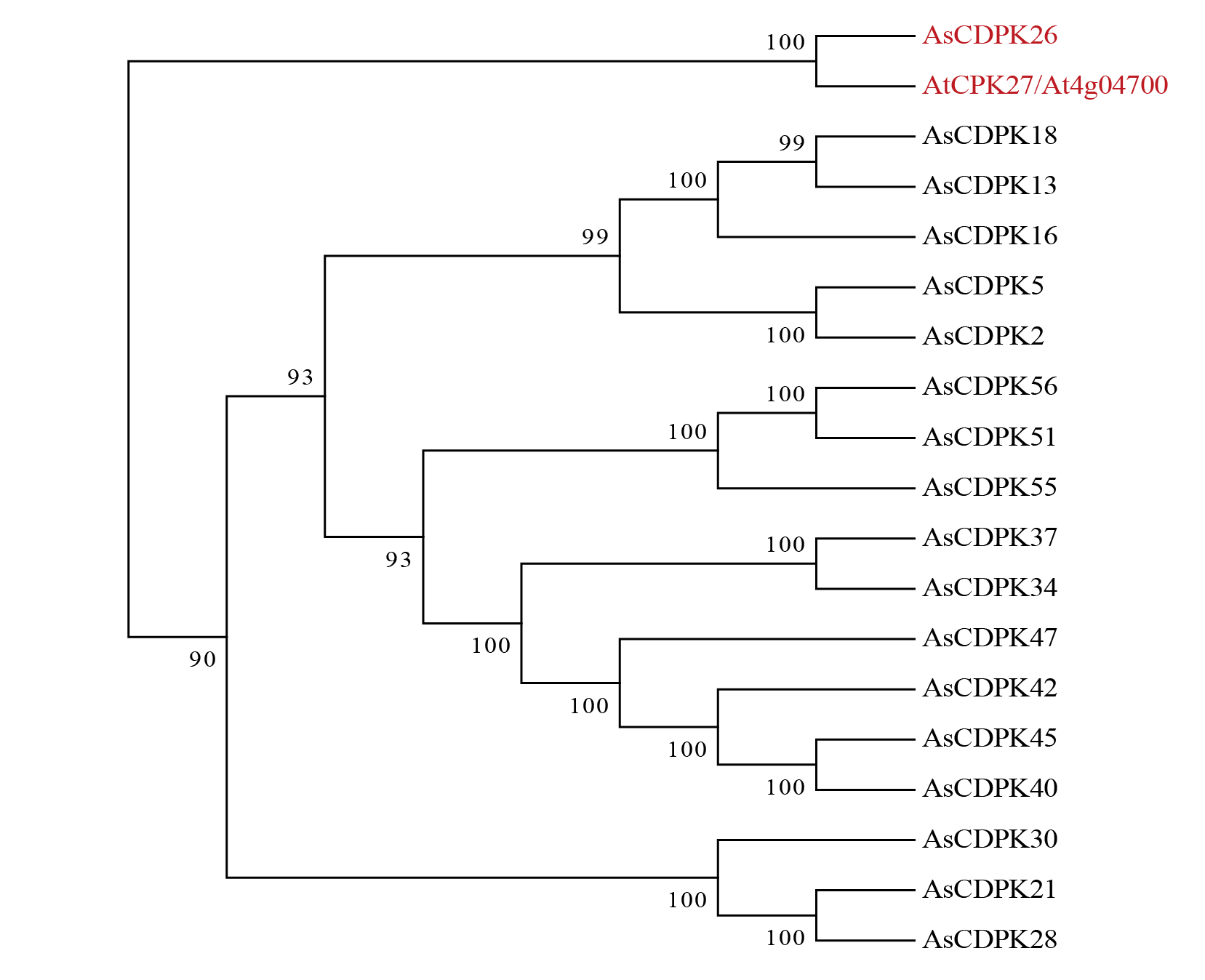

Supplement: Supplementary Figure 1 — Phylogenic tree of AsCDPKs and AtCPK27. [file Image1.png]
