# Supplementary material for: Identification and expression analysis of calcium-dependent protein kinase family in oat (Avena sativa L.) and their functions in response to saline-alkali stresses
Source: Front Plant Sci. 2024 Oct 10;15:1395696. doi: 10.3389/fpls.2024.1395696 (PMC11499199; doi:10.3389/fpls.2024.1395696)
Supplement: Supplementary file 2 [file Table1.docx]

**Table S1.** Primers used in qPCR analysis in this study.

| Primer name | Primer sequence (5′→3′) | Size (bp) |
| --- | --- | --- |
| *AsCDPK34-*F | CAACAAGAAAGAGAACTCGC | 20 |
| *AsCDPK34-*R | AACACCTTGTTCAGTCTCAG | 20 |
| *AsCDPK13-*F | TGCTGGTATTCTGAAGCAC | 19 |
| *AsCDPK13-*R | TGGTGCATTATCTGGATCTC | 20 |
| *AsCDPK40-*F | ATAAAGCAAATGTTCGATGTCA | 22 |
| *AsCDPK40-*R | TCTTAACATGGACAGACACG | 20 |
| *AsCDPK45-*F | TAAAGCAAATGTTCGATGTCA | 21 |
| *AsCDPK45-*R | TGAGAAGGCCTCTTGGA | 17 |
| *AsCDPK26-*F | ACACCTCCTGGTTACAACAAC | 21 |
| *AsCDPK26-*R  *As-actin-*F  *As-actin-*R | GATCTCGGTGCAGAGGTAAG  GCACAAGTGATGCCAGAATAGC  CGAGATGCATTAGATTCGTTGG | 20  22  22 |
| *AsCDPK26-*F1 | GGGTATAACAATCACGCGCA | 20 |
| *AsCDPK26-*R1  *AsCDPK26-*F2  *AsCDPK26-*R2 | ATCATGGTGCAGAACTCCTC  CTGAACGAGGAAGAGATCAA  TGAACCCGCTATTATCCTTG | 20  20  20 |
| *Cr-actin-*F  *Cr-actin-*R  *AsNHX1-*F  *AsNHX1-*R  *AsHKT1-*F  *AsHKT1-*R  *AsSOS1-*F  *AsSOS1-*R  *CrNHX1-*F  *CrNHX1-*R | ACATCAAGGAGAAGCTGT  TGTTGTTGTAGAGGTCCTTG  CTTAACAAAGAAGACGGCAC  CATCATGCCAAACAGCATAG  CATGCCATACAGCTTTTCTG  TCAGAAACACAGGAAGTAGC  GCATTCGTATATGGGCCG  AACCGTCGAGATGACCAC  CTGTTCGGCTACTACTACCT  ATCATGAGCGTGAAGAAGTT | 18  20  20  20  20  20  18  18  20  20 |

**Table S2.** RT-qPCR reaction system.

| Reagent | Dosage/μL |
| --- | --- |
| TB Green Premix Ex Taq Ⅱ (TliRNaseH Plus) (2×) | 5.0 |
| Primer-F | 0.2 |
| Primer-R | 0.2 |
| DNA | 0.5 |
| ddH_2_O | 4.1 |
| Total | 12.0 |

**Table S3.** Basic information on CDPK of *Arabidopsis*, rice and oat.

| Gene name | Gene ID | Species | Gene name | Gene ID | Species |
| --- | --- | --- | --- | --- | --- |
| *AtCDPK1* | At5g04870 | *Arabidopsis thaliana* | *OsCDPK30* | LOC_Os07g44710.1 | *Oryza sativa* |
| *AtCDPK2* | At3g10660 | *Arabidopsis thaliana* | *OsCDPK31* | AK110341 | *Oryza sativa* |
| *AtCDPK3* | At4g23650 | *Arabidopsis thaliana* | *AsCDPK1* | AVESA.00001b.r1.2Ag0000636.1 | *Avena sativa* |
| *AtCDPK4* | At4g09570 | *Arabidopsis thaliana* | *AsCDPK2* | AVESA.00001b.r1.4Ag0001693.1 | *Avena sativa* |
| *AtCDPK5* | At4g35310 | *Arabidopsis thaliana* | *AsCDPK3* | AVESA.00001b.r1.2Cg0002505.1 | *Avena sativa* |
| *AtCDPK6* | At2g17290 | *Arabidopsis thaliana* | *AsCDPK4* | AVESA.00001b.r1.6Cg0003063.1 | *Avena sativa* |
| *AtCDPK7* | At5g12480 | *Arabidopsis thaliana* | *AsCDPK5* | AVESA.00001b.r1.7Cg0000523.1 | *Avena sativa* |
| *AtCDPK8* | At5g19450 | *Arabidopsis thaliana* | *AsCDPK6* | AVESA.00001b.r1.7Cg0000520.1 | *Avena sativa* |
| *AtCDPK9* | At3g20410 | *Arabidopsis thaliana* | *AsCDPK7* | AVESA.00001b.r1.2Dg0001748.1 | *Avena sativa* |
| *AtCDPK10* | At1g18890 | *Arabidopsis thaliana* | *AsCDPK8* | AVESA.00001b.r1.2Dg0003120.1 | *Avena sativa* |
| *AtCDPK11* | At1g35670 | *Arabidopsis thaliana* | *AsCDPK9* | AVESA.00001b.r1.4Dg0002013.1 | *Avena sativa* |
| *AtCDPK12* | At5g23580 | *Arabidopsis thaliana* | *AsCDPK10* | AVESA.00001b.r1.6Dg0000850.1 | *Avena sativa* |
| *AtCDPK13* | At3g51850 | *Arabidopsis thaliana* | *AsCDPK11* | AVESA.00001b.r1.2Ag0001803.1 | *Avena sativa* |
| *AtCDPK14* | At2g41860 | *Arabidopsis thaliana* | *AsCDPK12* | AVESA.00001b.r1.4Ag0000684.1 | *Avena sativa* |
| *AtCDPK15* | At4g21940 | *Arabidopsis thaliana* | *AsCDPK13* | AVESA.00001b.r1.4Ag0002412.1 | *Avena sativa* |
| *AtCDPK16* | At2g17890 | *Arabidopsis thaliana* | *AsCDPK14* | AVESA.00001b.r1.5Ag0001479.1 | *Avena sativa* |
| *AtCDPK17* | At5g12180 | *Arabidopsis thaliana* | *AsCDPK15* | AVESA.00001b.r1.6Ag0001120.1 | *Avena sativa* |
| *AtCDPK18* | At4g36070 | *Arabidopsis thaliana* | *AsCDPK16* | AVESA.00001b.r1.4Cg0000976.1 | *Avena sativa* |
| *AtCDPK19* | At1g61950 | *Arabidopsis thaliana* | *AsCDPK17* | AVESA.00001b.r1.7Cg0001593.1 | *Avena sativa* |
| *AtCDPK20* | At2g38910 | *Arabidopsis thaliana* | *AsCDPK18* | AVESA.00001b.r1.1Dg0003388.1 | *Avena sativa* |
| *AtCDPK21* | At4g04720 | *Arabidopsis thaliana* | *AsCDPK19* | AVESA.00001b.r1.3Dg0001914.1 | *Avena sativa* |
| *AtCDPK22* | At4g04710 | *Arabidopsis thaliana* | *AsCDPK20* | AVESA.00001b.r1.4Dg0001000.1 | *Avena sativa* |
| *AtCDPK23* | At4g04740 | *Arabidopsis thaliana* | *AsCDPK21* | AVESA.00001b.r1.1Ag0003574.1 | *Avena sativa* |
| *AtCDPK24* | At2g31500 | *Arabidopsis thaliana* | *AsCDPK22* | AVESA.00001b.r1.1Ag0002101.1 | *Avena sativa* |
| *AtCDPK25* | At2g35890 | *Arabidopsis thaliana* | *AsCDPK23* | AVESA.00001b.r1.2Ag0000104.1 | *Avena sativa* |
| *AtCDPK26* | At4g38230 | *Arabidopsis thaliana* | *AsCDPK24* | AVESA.00001b.r1.3Ag0001628.1 | *Avena sativa* |
| *AtCDPK27* | At4g04700 | *Arabidopsis thaliana* | *AsCDPK25* | AVESA.00001b.r1.4Ag0002698.1 | *Avena sativa* |
| *AtCDPK28* | At5g66210 | *Arabidopsis thaliana* | *AsCDPK26* | AVESA.00001b.r1.2Cg0000672.1 | *Avena sativa* |
| *AtCDPK29* | At1g76040 | *Arabidopsis thaliana* | *AsCDPK27* | AVESA.00001b.r1.7Cg0001726.1 | *Avena sativa* |
| *AtCDPK30* | At1g74740 | *Arabidopsis thaliana* | *AsCDPK28* | AVESA.00001b.r1.1Dg0002135.1 | *Avena sativa* |
| *AtCDPK31* | At4g04695 | *Arabidopsis thaliana* | *AsCDPK29* | AVESA.00001b.r1.2Dg0002567.1 | *Avena sativa* |
| *AtCDPK32* | At3g57530 | *Arabidopsis thaliana* | *AsCDPK30* | AVESA.00001b.r1.3Dg0001187.1 | *Avena sativa* |
| *AtCDPK33* | At1g50700 | *Arabidopsis thaliana* | *AsCDPK31* | AVESA.00001b.r1.1Ag0002914.1 | *Avena sativa* |
| *AtCDPK34* | At5g19360 | *Arabidopsis thaliana* | *AsCDPK32* | AVESA.00001b.r1.1Ag0001427.1 | *Avena sativa* |
| *OsCDPK1* | LOC_Os01g43410.1 | *Oryza sativa* | *AsCDPK33* | AVESA.00001b.r1.2Ag0001721.1 | *Avena sativa* |
| *OsCDPK2* | LOC_Os01g59360.1 | *Oryza sativa* | *AsCDPK34* | AVESA.00001b.r1.3Ag0002268.1 | *Avena sativa* |
| *OsCDPK3* | LOC_Os01g61590.1 | *Oryza sativa* | *AsCDPK35* | AVESA.00001b.r1.4Ag0001825.1 | *Avena sativa* |
| *OsCDPK4* | LOC_Os02g03410.1 | *Oryza sativa* | *AsCDPK36* | AVESA.00001b.r1.2Cg0002423.1 | *Avena sativa* |
| *OsCDPK5* | LOC_Os02g46090.1 | *Oryza sativa* | *AsCDPK37* | AVESA.00001b.r1.3Cg0002463.1 | *Avena sativa* |
| *OsCDPK6* | LOC_Os02g58520.1 | *Oryza sativa* | *AsCDPK38* | AVESA.00001b.r1.1Dg0001468.1 | *Avena sativa* |
| *OsCDPK7* | LOC_Os03g03660.2 | *Oryza sativa* | *AsCDPK39* | AVESA.00001b.r1.2Dg0001661.1 | *Avena sativa* |
| *OsCDPK8* | LOC_Os03g59390.1 | *Oryza sativa* | *AsCDPK40* | AVESA.00001b.r1.4Dg0002141.1 | *Avena sativa* |
| *OsCDPK9* | LOC_Os03g48270.1 | *Oryza sativa* | *AsCDPK41* | AVESA.00001b.r1.4Ag0001166.1 | *Avena sativa* |
| *OsCDPK10* | LOC_Os03g57450.1 | *Oryza sativa* | *AsCDPK42* | AVESA.00001b.r1.4Ag0001829.1 | *Avena sativa* |
| *OsCDPK11* | LOC_Os03g57510.1 | *Oryza sativa* | *AsCDPK43* | AVESA.00001b.r1.6Ag0002165.1 | *Avena sativa* |
| *OsCDPK12* | LOC_Os04g47300.1 | *Oryza sativa* | *AsCDPK44* | AVESA.00001b.r1.2Cg0000500.1 | *Avena sativa* |
| *OsCDPK13* | LOC_Os04g49510.1 | *Oryza sativa* | *AsCDPK45* | AVESA.00001b.r1.7Cg0000381.1 | *Avena sativa* |
| *OsCDPK14* | LOC_Os05g41270.1 | *Oryza sativa* | *AsCDPK46* | AVESA.00001b.r1.7Cg0001047.1 | *Avena sativa* |
| *OsCDPK15* | LOC_Os05g50810.1 | *Oryza sativa* | *AsCDPK47* | AVESA.00001b.r1.2Dg0002401.1 | *Avena sativa* |
| *OsCDPK16* | LOC_Os05g39090.1 | *Oryza sativa* | *AsCDPK48* | AVESA.00001b.r1.3Dg0001812.1 | *Avena sativa* |
| *OsCDPK17* | LOC_Os07g06740.2 | *Oryza sativa* | *AsCDPK49* | AVESA.00001b.r1.4Dg0001473.1 | *Avena sativa* |
| *OsCDPK18* | LOC_Os07g22710.1 | *Oryza sativa* | *AsCDPK50* | AVESA.00001b.r1.5Dg0001148.1 | *Avena sativa* |
| *OsCDPK19* | LOC_Os07g33110.1 | *Oryza sativa* | *AsCDPK51* | AVESA.00001b.r1.1Ag0002412.1 | *Avena sativa* |
| *OsCDPK20* | LOC_Os07g38120.1 | *Oryza sativa* | *AsCDPK52* | AVESA.00001b.r1.5Ag0001519.1 | *Avena sativa* |
| *OsCDPK21* | LOC_Os08g42750.1 | *Oryza sativa* | *AsCDPK53* | AVESA.00001b.r1.5Ag0002848.1 | *Avena sativa* |
| *OsCDPK22* | LOC_Os09g33910.1 | *Oryza sativa* | *AsCDPK54* | AVESA.00001b.r1.5Cg0002481.1 | *Avena sativa* |
| *OsCDPK23* | LOC_Os10g39420.1 | *Oryza sativa* | *AsCDPK55* | AVESA.00001b.r1.6Cg0001428.1 | *Avena sativa* |
| *OsCDPK24* | LOC_Os11g07040.1 | *Oryza sativa* | *AsCDPK56* | AVESA.00001b.r1.2Dg0000664.1 | *Avena sativa* |
| *OsCDPK25* | LOC_Os11g04170.1 | *Oryza sativa* | *AsCDPK57* | AVESA.00001b.r1.5Dg0002397.1 | *Avena sativa* |
| *OsCDPK26* | LOC_Os12g03970.1 | *Oryza sativa* | *AsCDPK58* | AVESA.00001b.r1.3Ag0001659.1 | *Avena sativa* |
| *OsCDPK27* | LOC_Os12g30150.1 | *Oryza sativa* | *AsCDPK59* | AVESA.00001b.r1.3Cg0000800.1 | *Avena sativa* |
| *OsCDPK28* | LOC_Os12g07230.1 | *Oryza sativa* | *AsCDPK60* | AVESA.00001b.r1.3Dg0001210.1 | *Avena sativa* |
| *OsCDPK29* | LOC_Os12g12860.1 | *Oryza sativa* |  |  |  |

**Table S4.** Duplications of *AsCDPK* genes.

| Gene1 | Gene2 | *Ka* | *Ks* | *Ka/Ks* |
| --- | --- | --- | --- | --- |
| AVESA.00001b.r1.1Ag0001427.1 | AVESA.00001b.r1.1Ag0002914.1 | 0.004 | 0.068 | 0.059 |
| AVESA.00001b.r1.1Ag0002101.1 | AVESA.00001b.r1.1Ag0003574.1 | 0.007 | 0.138 | 0.048 |
| AVESA.00001b.r1.1Ag0001427.1 | AVESA.00001b.r1.1Dg0001468.1 | 0.002 | 0.040 | 0.040 |
| AVESA.00001b.r1.1Ag0002101.1 | AVESA.00001b.r1.1Dg0002135.1 | 0.001 | 0.057 | 0.014 |
| AVESA.00001b.r1.1Ag0002914.1 | AVESA.00001b.r1.1Dg0001468.1 | 0.004 | 0.065 | 0.061 |
| AVESA.00001b.r1.1Ag0003574.1 | AVESA.00001b.r1.1Dg0002135.1 | 0.007 | 0.138 | 0.054 |
| AVESA.00001b.r1.1Ag0002412.1 | AVESA.00001b.r1.2Dg0000664.1 | 0.000 | 0.017 | 0.000 |
| AVESA.00001b.r1.1Ag0003574.1 | AVESA.00001b.r1.3Ag0001628.1 | 0.115 | 0.869 | 0.132 |
| AVESA.00001b.r1.1Ag0001427.1 | AVESA.00001b.r1.3Ag0002268.1 | 0.049 | 0.652 | 0.075 |
| AVESA.00001b.r1.1Ag0002914.1 | AVESA.00001b.r1.3Ag0002268.1 | 0.054 | 0.694 | 0.077 |
| AVESA.00001b.r1.1Ag0002101.1 | AVESA.00001b.r1.3Ag0001628.1 | 0.186 | 1.021 | 0.182 |
| AVESA.00001b.r1.1Ag0001427.1 | AVESA.00001b.r1.3Cg0002463.1 | 0.050 | 0.635 | 0.079 |
| AVESA.00001b.r1.1Ag0002914.1 | AVESA.00001b.r1.3Cg0002463.1 | 0.055 | 0.669 | 0.083 |
| AVESA.00001b.r1.1Ag0001427.1 | AVESA.00001b.r1.3Dg0001812.1 | 0.046 | 0.633 | 0.073 |
| AVESA.00001b.r1.1Ag0002914.1 | AVESA.00001b.r1.3Dg0001812.1 | 0.050 | 0.670 | 0.074 |
| AVESA.00001b.r1.1Ag0003574.1 | AVESA.00001b.r1.3Dg0001187.1 | 0.114 | 0.858 | 0.133 |
| AVESA.00001b.r1.1Ag0002101.1 | AVESA.00001b.r1.3Dg0001187.1 | 0.118 | 0.917 | 0.128 |
| AVESA.00001b.r1.1Ag0002412.1 | AVESA.00001b.r1.6Cg0001428.1 | 0.006 | 0.078 | 0.076 |
| AVESA.00001b.r1.1Dg0002135.1 | AVESA.00001b.r1.3Ag0001628.1 | 0.117 | 0.955 | 0.123 |
| AVESA.00001b.r1.1Dg0001468.1 | AVESA.00001b.r1.3Ag0002268.1 | 0.048 | 0.634 | 0.075 |
| AVESA.00001b.r1.1Dg0001468.1 | AVESA.00001b.r1.3Cg0002463.1 | 0.049 | 0.629 | 0.078 |
| AVESA.00001b.r1.1Dg0001468.1 | AVESA.00001b.r1.3Dg0001812.1 | 0.045 | 0.615 | 0.073 |
| AVESA.00001b.r1.1Dg0002135.1 | AVESA.00001b.r1.3Dg0001187.1 | 0.116 | 0.943 | 0.123 |
| AVESA.00001b.r1.1Dg0003388.1 | AVESA.00001b.r1.4Ag0002412.1 | 0.003 | 0.039 | 0.083 |
| AVESA.00001b.r1.1Dg0003388.1 | AVESA.00001b.r1.4Cg0000976.1 | 0.011 | 0.086 | 0.131 |
| AVESA.00001b.r1.2Ag0001721.1 | AVESA.00001b.r1.2Cg0002423.1 | 0.019 | 0.100 | 0.190 |
| AVESA.00001b.r1.2Ag0001803.1 | AVESA.00001b.r1.2Cg0002505.1 | 0.010 | 0.089 | 0.116 |
| AVESA.00001b.r1.2Ag0000104.1 | AVESA.00001b.r1.2Cg0000672.1 | 0.006 | 0.122 | 0.046 |
| AVESA.00001b.r1.2Ag0001721.1 | AVESA.00001b.r1.2Dg0001661.1 | 0.007 | 0.042 | 0.164 |
| AVESA.00001b.r1.2Ag0001803.1 | AVESA.00001b.r1.2Dg0001748.1 | 0.002 | 0.028 | 0.083 |
| AVESA.00001b.r1.2Ag0000636.1 | AVESA.00001b.r1.2Dg0003120.1 | 0.001 | 0.010 | 0.074 |
| AVESA.00001b.r1.2Ag0000104.1 | AVESA.00001b.r1.2Dg0002567.1 | 0.001 | 0.006 | 0.145 |
| AVESA.00001b.r1.2Ag0000636.1 | AVESA.00001b.r1.4Ag0001693.1 | 0.201 | 1.834 | 0.110 |
| AVESA.00001b.r1.2Ag0000636.1 | AVESA.00001b.r1.4Dg0002013.1 | 0.195 | 1.819 | 0.107 |
| AVESA.00001b.r1.2Ag0001803.1 | AVESA.00001b.r1.6Ag0001120.1 | 0.081 | 0.775 | 0.104 |
| AVESA.00001b.r1.2Ag0001803.1 | AVESA.00001b.r1.6Cg0003063.1 | 0.077 | 0.797 | 0.097 |
| AVESA.00001b.r1.2Ag0001803.1 | AVESA.00001b.r1.6Dg0000850.1 | 0.079 | 0.770 | 0.102 |
| AVESA.00001b.r1.2Ag0000636.1 | AVESA.00001b.r1.7Cg0000520.1 | 0.120 | 0.788 | 0.152 |
| AVESA.00001b.r1.2Cg0002423.1 | AVESA.00001b.r1.2Dg0001661.1 | 0.018 | 0.089 | 0.199 |
| AVESA.00001b.r1.2Cg0002505.1 | AVESA.00001b.r1.2Dg0001748.1 | 0.008 | 0.092 | 0.086 |
| AVESA.00001b.r1.2Cg0000500.1 | AVESA.00001b.r1.2Dg0002401.1 | 0.012 | 0.093 | 0.132 |
| AVESA.00001b.r1.2Cg0000672.1 | AVESA.00001b.r1.2Dg0002567.1 | 0.005 | 0.122 | 0.040 |
| AVESA.00001b.r1.2Cg0000500.1 | AVESA.00001b.r1.6Ag0002165.1 | 0.008 | 0.090 | 0.090 |
| AVESA.00001b.r1.2Cg0002505.1 | AVESA.00001b.r1.6Ag0001120.1 | 0.083 | 0.776 | 0.107 |
| AVESA.00001b.r1.2Cg0002505.1 | AVESA.00001b.r1.6Cg0003063.1 | 0.079 | 0.781 | 0.101 |
| AVESA.00001b.r1.2Cg0002505.1 | AVESA.00001b.r1.6Dg0000850.1 | 0.089 | 0.766 | 0.116 |
| AVESA.00001b.r1.2Dg0002401.1 | AVESA.00001b.r1.6Ag0002165.1 | 0.004 | 0.025 | 0.162 |
| AVESA.00001b.r1.2Dg0001748.1 | AVESA.00001b.r1.6Ag0001120.1 | 0.087 | 0.780 | 0.111 |
| AVESA.00001b.r1.2Dg0000664.1 | AVESA.00001b.r1.6Cg0001428.1 | 0.004 | 0.084 | 0.050 |
| AVESA.00001b.r1.2Dg0001748.1 | AVESA.00001b.r1.6Cg0003063.1 | 0.077 | 0.804 | 0.096 |
| AVESA.00001b.r1.2Dg0001748.1 | AVESA.00001b.r1.6Dg0000850.1 | 0.080 | 0.777 | 0.103 |
| AVESA.00001b.r1.2Dg0003120.1 | AVESA.00001b.r1.7Cg0000520.1 | 0.119 | 0.774 | 0.154 |
| AVESA.00001b.r1.3Ag0002268.1 | AVESA.00001b.r1.3Cg0002463.1 | 0.002 | 0.084 | 0.029 |
| AVESA.00001b.r1.3Ag0001659.1 | AVESA.00001b.r1.3Cg0000800.1 | 0.028 | 0.107 | 0.260 |
| AVESA.00001b.r1.3Ag0001628.1 | AVESA.00001b.r1.3Dg0001187.1 | 0.000 | 0.006 | 0.000 |
| AVESA.00001b.r1.3Ag0001659.1 | AVESA.00001b.r1.3Dg0001210.1 | 0.006 | 0.069 | 0.086 |
| AVESA.00001b.r1.3Ag0002268.1 | AVESA.00001b.r1.3Dg0001812.1 | 0.000 | 0.043 | 0.000 |
| AVESA.00001b.r1.3Cg0002463.1 | AVESA.00001b.r1.3Dg0001812.1 | 0.003 | 0.085 | 0.030 |
| AVESA.00001b.r1.3Cg0000800.1 | AVESA.00001b.r1.3Dg0001210.1 | 0.021 | 0.114 | 0.184 |
| AVESA.00001b.r1.3Dg0001914.1 | AVESA.00001b.r1.4Ag0002698.1 | 0.001 | 0.031 | 0.027 |
| AVESA.00001b.r1.4Ag0002412.1 | AVESA.00001b.r1.4Cg0000976.1 | 0.010 | 0.086 | 0.122 |
| AVESA.00001b.r1.4Ag0000684.1 | AVESA.00001b.r1.4Dg0001000.1 | 0.001 | 0.020 | 0.042 |
| AVESA.00001b.r1.4Ag0001166.1 | AVESA.00001b.r1.4Dg0001473.1 | 0.004 | 0.066 | 0.057 |
| AVESA.00001b.r1.4Ag0001693.1 | AVESA.00001b.r1.4Dg0002013.1 | 0.004 | 0.094 | 0.045 |
| AVESA.00001b.r1.4Ag0001825.1 | AVESA.00001b.r1.4Dg0002141.1 | 0.002 | 0.045 | 0.036 |
| AVESA.00001b.r1.4Ag0000684.1 | AVESA.00001b.r1.7Cg0001593.1 | 0.005 | 0.056 | 0.092 |
| AVESA.00001b.r1.4Ag0001166.1 | AVESA.00001b.r1.7Cg0001047.1 | 0.009 | 0.146 | 0.060 |
| AVESA.00001b.r1.4Ag0001825.1 | AVESA.00001b.r1.7Cg0000381.1 | 0.006 | 0.121 | 0.047 |
| AVESA.00001b.r1.4Dg0001473.1 | AVESA.00001b.r1.7Cg0001047.1 | 0.008 | 0.141 | 0.057 |
| AVESA.00001b.r1.4Dg0002141.1 | AVESA.00001b.r1.7Cg0000381.1 | 0.006 | 0.118 | 0.049 |
| AVESA.00001b.r1.4Dg0001000.1 | AVESA.00001b.r1.7Cg0001593.1 | 0.004 | 0.059 | 0.073 |
| AVESA.00001b.r1.5Ag0002848.1 | AVESA.00001b.r1.5Cg0002481.1 | 0.018 | 0.075 | 0.246 |
| AVESA.00001b.r1.5Ag0002848.1 | AVESA.00001b.r1.5Dg0002397.1 | 0.002 | 0.016 | 0.100 |
| AVESA.00001b.r1.5Ag0001519.1 | AVESA.00001b.r1.5Dg0001148.1 | 0.002 | 0.015 | 0.151 |
| AVESA.00001b.r1.5Cg0002481.1 | AVESA.00001b.r1.5Dg0002397.1 | 0.020 | 0.075 | 0.267 |
| AVESA.00001b.r1.6Ag0001120.1 | AVESA.00001b.r1.6Cg0003063.1 | 0.009 | 0.067 | 0.131 |
| AVESA.00001b.r1.6Ag0001120.1 | AVESA.00001b.r1.6Dg0000850.1 | 0.001 | 0.023 | 0.034 |
| AVESA.00001b.r1.6Cg0003063.1 | AVESA.00001b.r1.6Dg0000850.1 | 0.009 | 0.053 | 0.166 |
